# Simultaneous induction of mutant alleles of two allergenic genes in soybean by using site-directed mutagenesis

**DOI:** 10.1186/s12870-020-02708-6

**Published:** 2020-11-11

**Authors:** Shota Sugano, Aya Hirose, Yuhei Kanazashi, Kohei Adachi, Miki Hibara, Takeshi Itoh, Masafumi Mikami, Masaki Endo, Sakiko Hirose, Nobuyuki Maruyama, Jun Abe, Tetsuya Yamada

**Affiliations:** 1 Graduate School of Agriculture, Hokkaido University, Kita 9, Nishi 9, Kita-ku, Sapporo, Hokkaido 060-8589 Japan; 2 Bioinformatics Team, Advanced Analysis Center, National Agricultural and Food Research Organization, 2-1-2 Kannondai, Tsukuba, Ibaraki 305-8602 Japan; 3 Plant Genome Engineering Research Unit, Institute of Agrobiological Sciences, National Agricultural and Food Research Organization, 1-2, Owashi, Tsukuba, Ibaraki 305-8634 Japan; 4 Graduate School of Agriculture, Kyoto University, Uji, Kyoto, 611-0011 Japan

**Keywords:** CRISPR/Cas9, *Glycine max*, Gly m Bd 28 K, Gly m Bd 30 K, Hypoallergenic soybean

## Abstract

**Background:**

Soybean (*Glycine max*) is a major protein crop, because soybean protein has an amino acid score comparable to that of beef and egg white. However, many allergens have been identified among soybean proteins. A decrease in allergenic protein levels would be useful for expanding the market for soybean proteins and processed foods. Recently, the CRISPR/Cas9 system has been adopted as a powerful tool for the site-directed mutagenesis in higher plants. This system is expected to generate hypoallergenic soybean varieties.

**Results:**

We used two guide RNAs (gRNAs) and *Agrobacterium*-mediated transformation for simultaneous site-directed mutagenesis of two genes encoding the major allergens Gly m Bd 28 K and Gly m Bd 30 K in two Japanese soybean varieties, Enrei and Kariyutaka. We obtained two independent T_0_ Enrei plants and nine T_0_ Kariyutaka plants. Cleaved amplified polymorphic sequence (CAPS) analysis revealed that mutations were induced in both targeted loci of both soybean varieties. Sequencing analysis showed that deletions were the predominant mutation type in the targeted loci. The *Cas9*-free plants carrying the mutant alleles of the targeted loci with the transgenes excluded by genetic segregation were obtained in the T_2_ and T_3_ generations. Variable mutational spectra were observed in the targeted loci even in T_2_ and T_3_ progenies of the same T_0_ plant. Induction of multiple mutant alleles resulted in six haplotypes in the *Cas9*-free mutants derived from one T_0_ plant. Immunoblot analysis revealed that no Gly m Bd 28 K or Gly m Bd 30 K protein accumulated in the seeds of the *Cas9*-free plants. Whole-genome sequencing confirmed that a *Cas9*-free mutant had also no the other foreign DNA from the binary vector. Our results demonstrate the applicability of the CRISPR/Cas9 system for the production of hypoallergenic soybean plants.

**Conclusions:**

Simultaneous site-directed mutagenesis by the CRISPR/Cas9 system removed two major allergenic proteins from mature soybean seeds. This system enables rapid and efficient modification of seed components in soybean varieties.

**Supplementary Information:**

The online version contains supplementary material available at 10.1186/s12870-020-02708-6.

## Background

Soybean (*Glycine max*, 2*n* = 2*x* = 40) is one of the most important protein crops used for food and forage worldwide, because its seeds contain high-quality proteins with an amino acid score comparable to that of beef and egg white [1]. Diverse soybean proteins are responsible for the physical properties of foods and other products made from soybean seeds [2, 3]. In the USA and Europe, 5 to 8% of babies and 2% of adults are allergic to soybean [4]. Several subunits of major storage proteins such as 7S and 11S globulins and 2S albumin are representative soybean allergens [5]. The vicilin-like glycoprotein Gly m Bd 28 K and the oil-body-associated protein Gly m Bd 30 K are also reported as major soybean allergens [6, 7]. Hydrophobic proteins Gly m 1A and Gly m 1B and the hull protein Gly m 2 are related to asthma outbreaks in Spain [8, 9]. Profilin Gly m 3 and the pathogenesis-related protein Gly m 4 are cross-reactive with antigens from other sources involved in sensitization and symptom induction [10, 11]. Positive response to soybean protein in allergic reaction has been reported in 14% of patients diagnosed with food allergies with atopic dermatitis [12]. Therefore, development of hypoallergenic soybean varieties or establishment of a procedure to remove allergens would be useful for expanding the market of soybean proteins and processed foods.

Protein fractionation on the basis of the differences in protein solubility at different salt concentrations and pH can be used to characterize the biochemical and physical properties of proteins [13–15]. This technique is also used for the removal of specific allergens from soy foods. Gly m Bd 30 K was efficiently removed from soy milk by acidifying it to pH 4.5 with 1 M Na_2_SO_4_ [16].

Genetic improvement of soybean is achieved by crossing plants carrying allergen-deficient alleles from soybean genetic resources or by mutagenesis to generate allergen-deficient mutant alleles. A number of spontaneous or induced mutants deficient in subunits of 7S or 11S globulins have been reported [17–20]. Among the germplasm of wild soybean (*G. soja*), Hajika et al. [20] found one accession lacking the α-, α’-, and β-subunits of 7S globulin. The deficiency of these subunits is controlled by a single dominant gene (*Scg-1*), which is closely associated with post-transcriptional gene silencing [21]. To develop hypoallergenic soybean through crossing and subsequent back-crossing, this dominant gene has been introduced into an elite variety, Fukuyutaka [22]. The soybean variety Yumeminori lacks α- and α’-subunits of 7S globulin, and Gly m Bd 28 K, and has a decreased level of the β-subunit of 7S globulin; this variety has been developed through mutagenesis by gamma-ray irradiation [23]. Mutagenesis of the soybean variety VLSoy-2 by gamma-ray irradiation generated mutant lines lacking the A3-subunit of 11S globulin [24]. This mutagenesis also produced plants lacking α- and α’-subunits of 7S globulin [24]. Stacking of recessive mutant alleles of the genes for Kunitz trypsin inhibitor, agglutinin, and Gly m Bd 30 K was performed in the genetic background of the soybean variety Williams 82 [25]. Proteome analysis revealed that the stacking of these mutant alleles markedly decreased the accumulation of these allergens [25].

The biotechnological approach can also help to decrease the accumulation of allergens in soybean seeds. Down-regulation of the gene encoding Gly m Bd 30 K greatly suppresses the accumulation of the targeted protein in seeds of transgenic soybean [26]. The accumulation of α-, α’-, and β-subunits of 7S globulin in soybean seeds can be greatly decreased through RNA interference or artificial microRNA systems [27, 28]. Recently, the transcription activator-like effector nucleases (TALENs) and clustered regularly interspaced short palindromic repeats (CRISPR)/CRISPR-associated endonuclease 9 (Cas9) systems have become the main platforms for site-directed mutagenesis in higher plants [29–32]. They enable the fine tuning of traits in soybean breeding when applied to various soybean varieties. The CRISPR/Cas9 system can be used to develop hypoallergenic soybean directly from elite varieties, because it has been optimized for various soybean varieties [33–37].

The subunits of 7S and 11S globulins are closely associated with seed characteristics important for food processing such as gel-forming and emulsifying properties [38–40]. To produce hypoallergenic soybeans without impairing the processing properties, we focused on two allergenic proteins, Gly m Bd 28 K and Gly m Bd 30 K, because no pyramiding of mutant alleles of these allergens in soybean has been reported. Here, we constructed a plasmid for simultaneous site-directed mutagenesis of these genes with the CRISPR/Cas9 system and used it for *Agrobacterium*-mediated transformation of two soybean varieties. *Cas9*-free plants carrying mutant alleles of the targeted loci, with the transgenes excluded by genetic segregation, were obtained in the T_2_ or T_3_ generations. Immunoblot analysis revealed that Gly m Bd 28 K and Gly m Bd 30 K proteins did not accumulate in seeds of the *Cas9*-free plants. Our results demonstrate the applicability of the CRISPR/Cas9 system for the production of hypoallergenic soybean plants.

## Results

Generation of transgenic soybean plants harboring the CRISPR/Cas9 expression module.

**Fig. 1:**
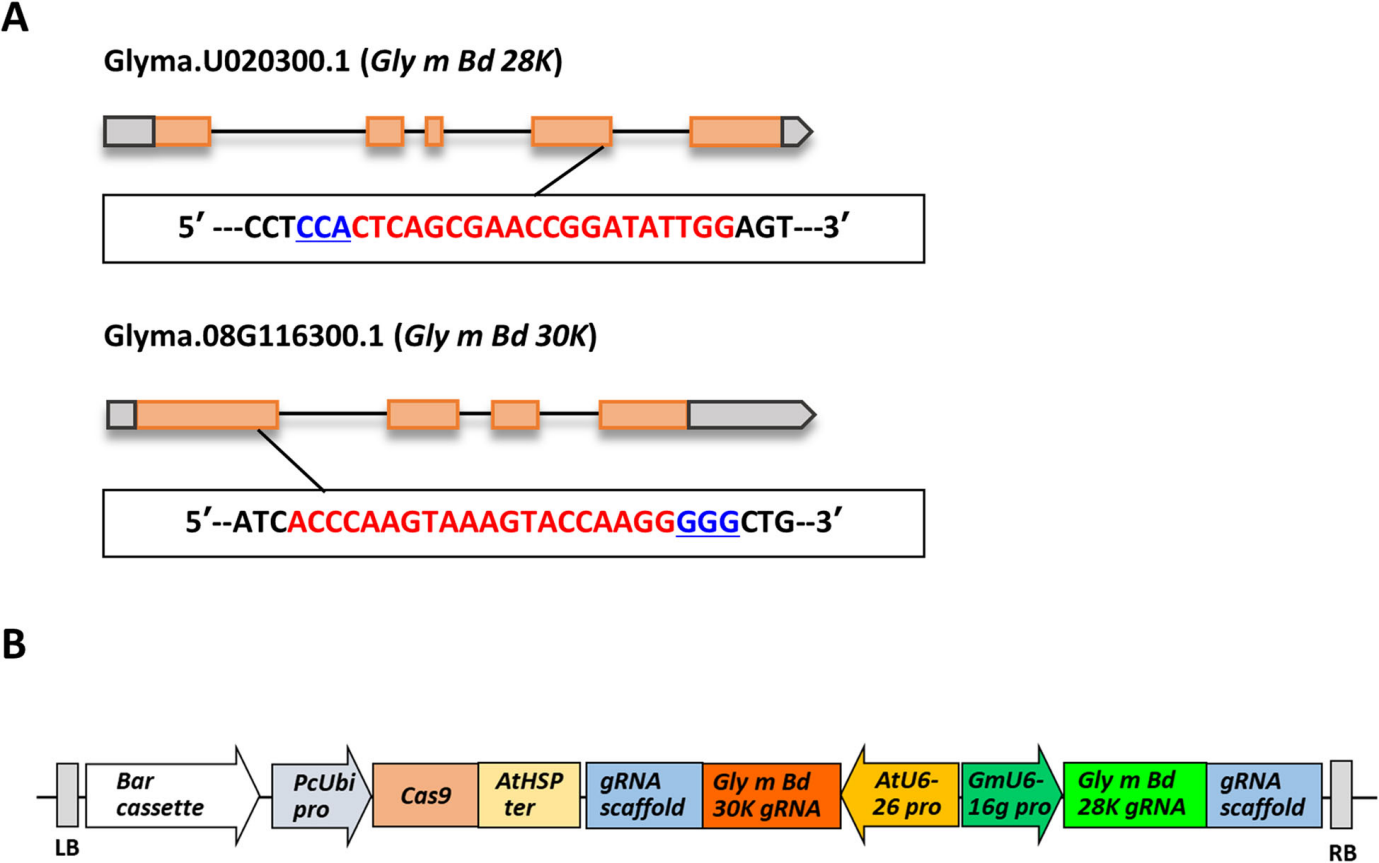
Targeted regions (**a**) and expression vector (**b**) of simultaneous site-directed mutagenesis of soybean genes *Gly m Bd 28 K* and *Gly m Bd 30 K* by using the CRISPR/Cas9 system. **a** Locations of the targeted sites of *Gly m Bd 28 K* and *Gly m Bd 30 K* genes. Boxes and pentagons, exons of the genes; bold lines, introns of the genes. Red and blue nucleotide sequences denote the gRNAs-targeted regions and the proto-spacer adjacent motif (PAM) regions, respectively. **b** Vector structure of the CRISPR/Cas9 expression module for soybean transformation. *Bar* cassette, *Bar*-marker gene unit; *PcUbi pro*, parsley (*Petroselinum crispum*) ubiquitin promoter; *AtHSP ter*, terminator of an Arabidopsis heat shock protein gene; *AtU6 pro*, Arabidopsis U6 promoter; *GmU6–16 g pro*, soybean U6–16 promoter

To conduct the site-directed mutagenesis of soybean with the CRISPR/Cas9 system, we designed two guide RNAs (gRNAs) to mutagenize the *Gly m Bd 28 K* and *Gly m Bd 30 K* loci (Fig. 1a). Explants of Enrei and Kariyutaka were inoculated with *Agrobacterium* harboring the pMR284_28K_30K plasmid (Fig. 1b). Two Enrei T_0_ plants (E1 and E2) and nine Kariyutaka T_0_ plants (K1 to K10) were obtained. All the T_0_ plants set T_1_ seeds. In our previous study, many T_0_ plants produced by our soybean transformation system failed to transmit the transgenes into the T_1_ progeny [41]. This fact indicates that T_0_ plants originated from chimeric tissues which contained transformed and non-transformed cells [41]. Therefore, we did not examine transgene integration or the induction of mutagenesis in the T_0_ plants, and grew representative T_1_ plants for further analyses. The plant numbers of the T_1_ and T_2_ generations were indicated by giving the T_0_ individual number followed by the branch number for each generation.

### Mutations in T_1_ plants detected by cleaved amplified polymorphic sequence (CAPS) analysis

**Fig. 2:**
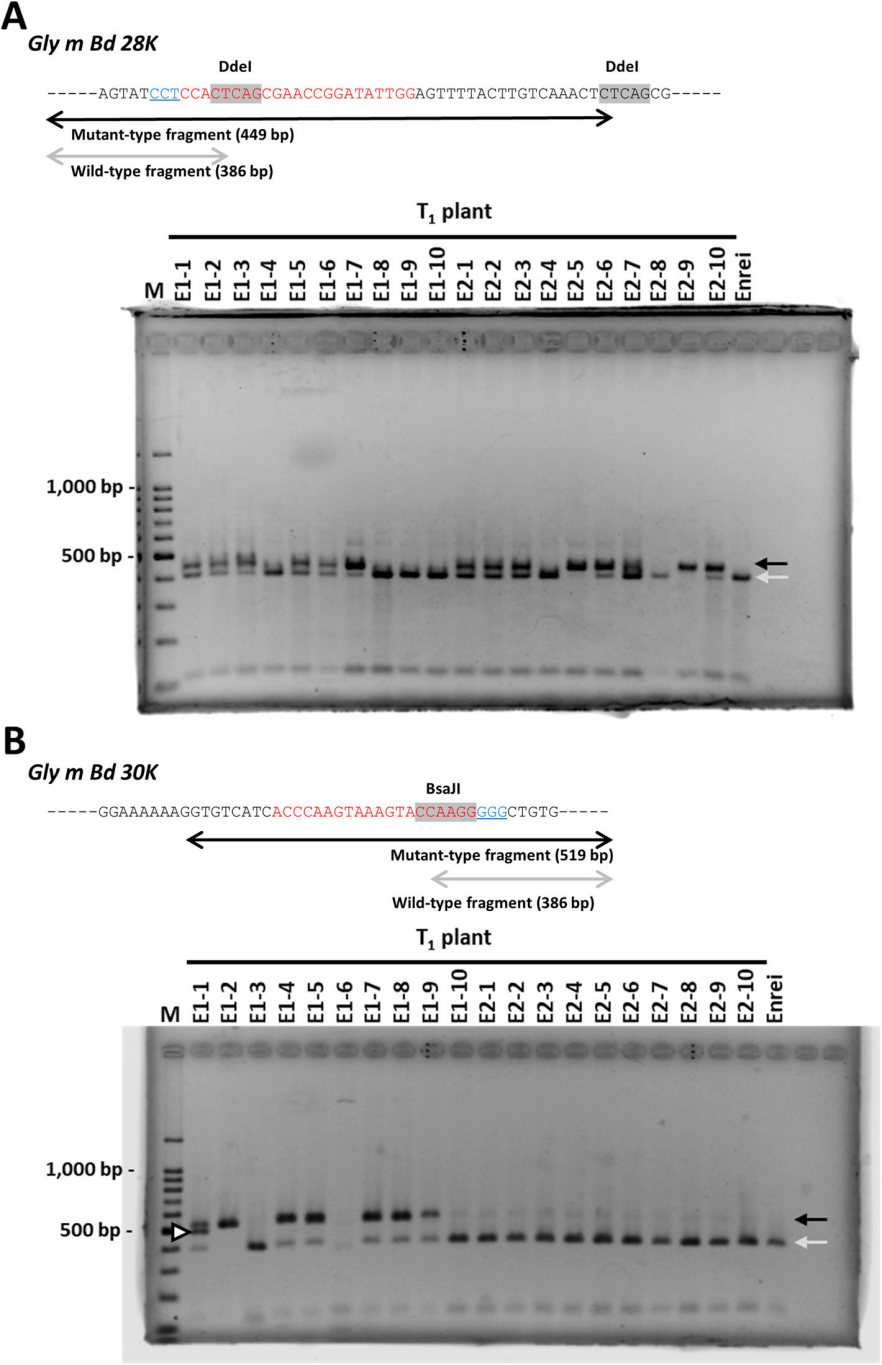
Confirmation of mutagenesis of targeted loci in representative Enrei-T_1_ plants by CAPS analysis. The schematic diagrams above panels show DdeI or BsaJI restriction sites (shaded in gray) in fragments amplified with specific primers. Red and blue nucleotide sequences have the same meaning as those in Fig. 1. Gray arrows, the sizes of expected wild-type fragments; black arrows, the sizes of expected mutant-type fragments; open triangle, a fragment of unexpected size considered as mutant type. M, molecular weight marker (100-bp ladder)

Representative 20 T_1_ Enrei plants and 25 T_1_ Kariyutaka plants derived from 12 T_0_ plants were grown, and the induction of mutagenesis in the targeted loci was evaluated by CAPS analysis of the genomic DNA. The DNA fragments were classed into wild-type and mutant-type based on the expected size; a fragment of unexpected size was also detected and considered as mutant-type (Fig. 2). Mutations were detected in both targeted loci in plants of both varieties (Fig. 2, Additional file 2: Figure S1, Additional file 1: Tables S1, S2). Integration of the transgene was also examined by PCR analysis with *Cas9*-specific primers. PCR analysis revealed that 11 T_1_ plants (24.4% of all T_1_ plants examined) were *Cas9-*free; among these, E1–4, E1–8, and E1–9 had mutant alleles in the *Gly m Bd 30 K* locus, whereas the others had the wild-type alleles of both targeted loci (Additional file 1: Tables S1, S2).

### Transmission of mutations and transgenes to the T_2_ generation

Because none of the *Cas9*-free T_1_ plants had mutations in both targeted loci, 13 representative T_1_ plants were advanced to the next generation. A total of 348 T_2_ seeds collected from the 13 T_1_ plants were evaluated for the mutations in the targeted loci (Table 1). In CAPS analysis, 227 (65%) T_2_ seeds showed mutant-type fragments of both targeted loci (Table 1, Additional file 2: Figs. S2, S3). No mutations were detected in 20 T_2_ seeds (Table 1). Thus, frequency of simultaneous site-directed mutagenesis in both targeted loci was much higher in the T_2_ generation than in the T_1_ generation (Table 1, Additional file 1: Tables S1, S2).

**Table 1:** Frequency of simultaneous site-directed mutagenesis in the targeted loci *Gly m Bd 28 K* and *Gly m Bd 30 K* in T_2_ seeds

T_1_ plant number^a^	Number of T_2_ seeds				
	Mutation			No mutation	Total
	*Gly m Bd 28 K* and *Gly m Bd 30 K*	*Gly m Bd 28 K* only	*Gly m Bd 30 K* only		
E1–1	18	11	8	2	39
E1–2	22	0	3	0	25
E1–5	9	4	3	1	17
E2–1	0	22	0	10	32
E2–2	1	20	0	5	26
K1–1	12	1	0	0	13
K1–3	16	3	4	2	25
K2–1	17	3	3	0	23
K2–2	20	7	4	0	31
K4–1	35	0	2	0	37
K5–1	50	0	0	0	50
K5–2	14	1	0	0	15
K6–1	12	3	0	0	15

The presence of mutations was evaluated by CAPS analysis

^a^The letter and first number correspond to the number of the parental T_0_ plant

**Fig. 3:**
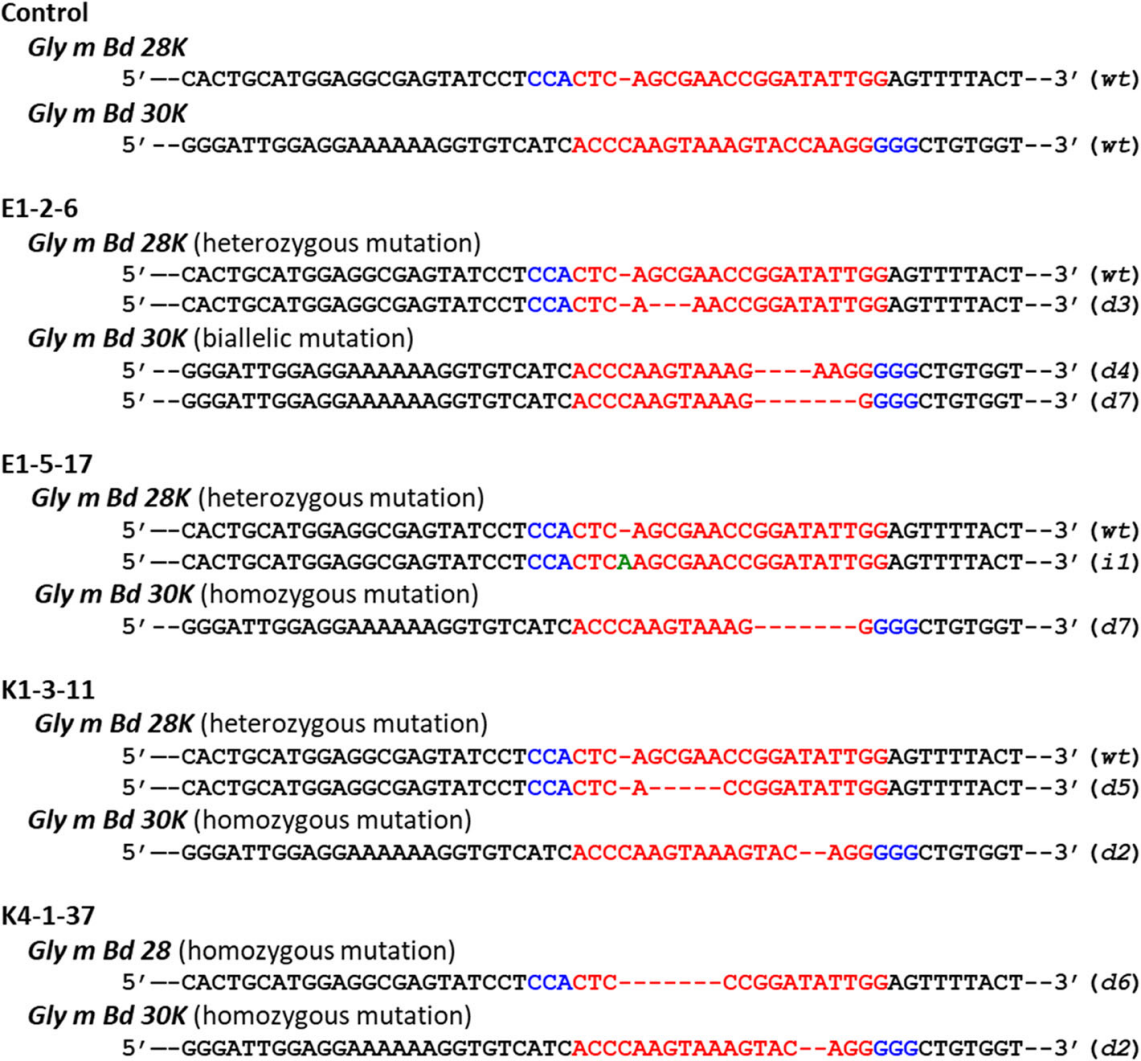
Mutational spectra of the targeted loci in double-mutant T_2_ seeds. Red and blue nucleotide sequences have the same meaning as those in Fig. 1. Green nucleotide denotes an insertion. Letters and numbers in parentheses indicate the type of mutation in the targeted locus: e.g., *d1*, a single-nucleotide deletion; *i1*, a single-nucleotide insertion; *wt*, no mutation. Control, reference sequence (Enrei or Kariyutaka)

Although the *Cas9* gene was detected in all 13 T_1_ plants (Additional file 1: Tables S1, S2), it was removed by genetic segregation in 52 T_2_ seeds (Table 2, Additional file 2: Fig. S4). Among them, 38 T_2_ seeds had mutant alleles in one or both targeted loci (Table 2). Of these 38 seeds, 14 seeds belonging to both varieties had mutant alleles in both targeted loci (double-mutants; Table 2). Representative mutational spectra of the T_2_ seeds are shown in Fig. 3. The genotypes were divided into homozygous (the *Gly m Bd 28 K* locus in K4–1-37 and *Gly m Bd 30 K* locus in E1–5-17, K1–3-11, and K4–1-37 T_2_ seeds), heterozygous (the *Gly m Bd 28 K* locus in E1–2-6 and E1–5-17, and K1–3-11 T_2_ seeds), and biallelic mutant types (the *Gly m Bd 30 K* locus in E1–2-6) (Fig. 3). Deletions were predominant in the mutational spectra of these T_2_ seeds (Fig. 3).

**Table 2:** Frequency of *Cas9*-positive and *Cas9*-free T_2_ seeds

T_1_ plant number^a^	*Cas9* integration	Number of T_2_ seeds				
		Mutation			No mutation	Total
		*Gly m Bd 28 K* and *Gly m Bd 30 K*	*Gly m Bd 28 K* only	*Gly m Bd 30 K* only		
E1–1	positive	16	10	3	0	29
	free	2	1	5	2	10
E1–2	positive	21	0	0	0	21
	free	1	0	3	0	4
E1–5	positive	8	4	0	1	13
	free	1	0	3	0	4
E2–1	positive	0	20	0	5	25
	free	0	2	0	5	7
E2–2	positive	1	20	0	0	21
	free	0	0	0	5	5
K1–1	positive	9	1	0	0	10
	free	3	0	0	0	3
K1–3	positive	13	3	0	0	16
	free	3	0	4	2	9
K2–1	positive	16	3	1	0	20
	free	1	0	2	0	3
K2–2	positive	19	7	0	0	26
	free	1	0	4	0	5
K4–1	positive	33	0	2	0	35
	free	2	0	0	0	2
K5–1	positive	50	0	0	0	50
	free	0	0	0	0	0
K5–2	positive	14	1	0	0	15
	free	0	0	0	0	0
K6–1	positive	12	3	0	0	15
	free	0	0	0	0	0

^a^The letter and first number correspond to the number of the parental T_0_ plant

### Development of double-mutant T_3_ seeds

We used the heterozygous and biallelic mutants to develop more homozygous mutant alleles. We collected T_3_ seeds and sequenced both targeted loci. In total, 4 haplotypes in Enrei and 21 haplotypes in Kariyutaka were found in the double-mutants (Table 3). Deletions (1 to 43 nucleotides) were the most common mutations (Table 3, Additional file 2: Figure S5). Predicted amino acid sequences of Gly m Bd 28 K (Figure S6) and Gly m Bd 30 K (Figure S7) are shown in Additional file 2. Three mutant alleles (*d3* and *d6* for the *Gly m Bd 28 K* locus, and *d6* for the *Gly m Bd 30 K* locus) had in-frame mutations (Additional file 2: Figures S6, S7). In the Gly m Bd 30 K locus, the 3-nucleotide deletion generated a stop codon at the mutation site, and the 33-nucleotide deletion was not predicted as an in-frame mutation, because the deleted region contained the splicing site (Additional file 2: Figure S7).

**Table 3:** Mutational spectra in double-mutant T_3_ plants

Donor plant	Haplotype	*Gly m Bd 28 K* locus	*Gly m Bd 30 K* locus
Enrei	E-type1	*i1*, *i1*	*d7*, *d7*
	E-type2	*d3*, *d3*	*d33*, *d33*
	E-type3	*d3*, *d3*	*d7*, *d7*
	E-type4	*d3*, *d3*	*d4*, *d4*
Kariyutaka	K-type1	*i1*, *i1*	*d5*, *d5*
	K-type2	*d5*, *d5*	*d2*, *d2*
	K-type3	*d5, wt*^a^	*d2*, *d2*
	K-type4	*d5*, *d5*	*d1*, *d1*
	K-type5	*d5*, *d5*	*d6*, *d6*
	K-type6	*d5*, *d5*	*d1, d6*^b^
	K-type7	*d5*, *d5*	*d1s1*, *d1s1*
	K-type8	*d5*, *d5*	*d1s1, wt*^a^
	K-type9	*d2*, *d2*	*d1s1*, *d1s1*
	K-type10	*d2*, *d2*	*d1s1, wt*^a^
	K-type11	*d2, d5*^b^	*d1s1*, *d1s1*
	K-type12	*d2, d5*^b^	*d1s1, wt*^a^
	K-type13	*d2, d5*^b^	*d1*, *d1*
	K-type14	*i1*, *i1*	*d43*, *d43*
	K-type15	*i1*, *i1*	*d1*, *d1*
	K-type16	*i1*, *i1*	*d1, d43*^b^
	K-type17	*i1, wt*^a^	*d1, d43*^b^
	K-type18	*d6*, *d6*	*d3*, *d3*
	K-type19	*i1*, *i1*	*d2*, *d2*
	K-type20	*i1*, *i1*	*d2, d1*^b^
	K-type21	*d6*, *d6*	*d2*, *d2*

Letters and numbers denote the alleles in the targeted loci: e.g., *d1*, a single-nucleotide deletion; *i1*, a single-nucleotide insertion; *d1s1*, a single-nucleotide deletion and substitution; *wt*, wild type

^a^Heterozygous mutation in the targeted locus

^b^Biallelic mutations in the targeted locus

### Analysis of Gly m Bd 28 K and Gly m Bd 30 K proteins in mature double-mutant seeds

**Fig. 4:**
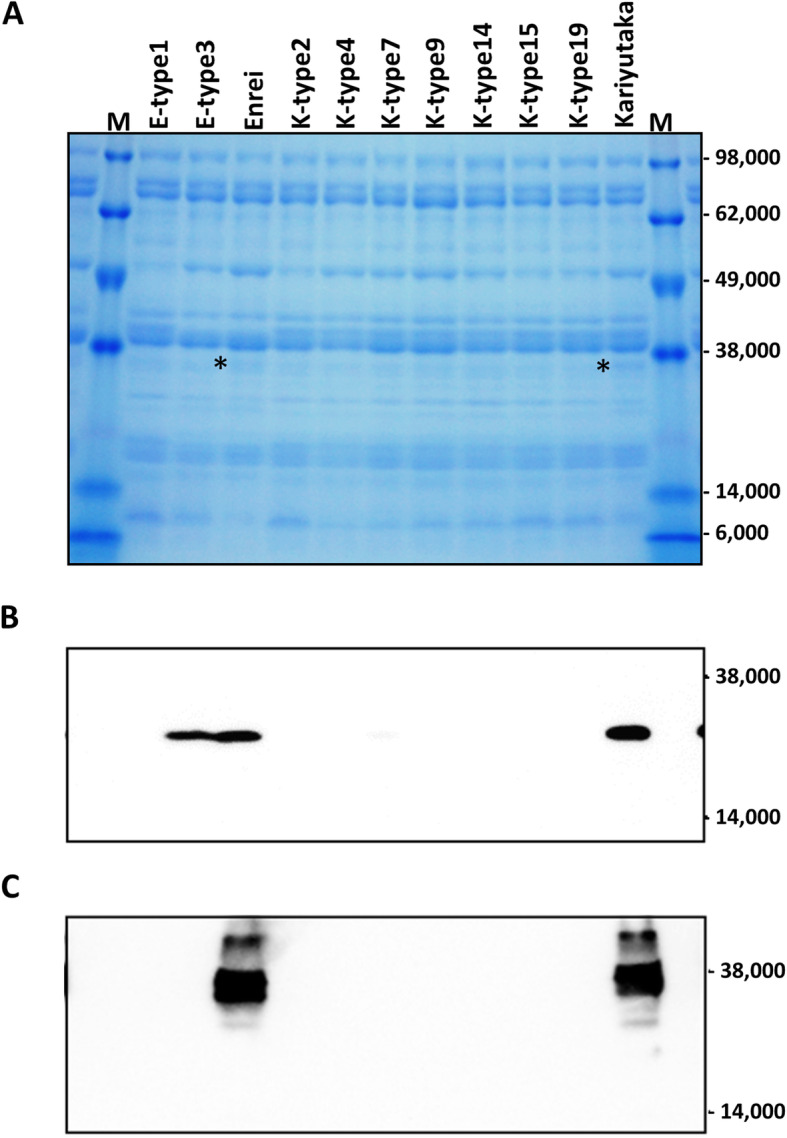
SDS-PAGE and immunoblot analyses of the crude proteins of representative double-mutant T_3_ and wild-type mature seeds. **a** Proteins separated by SDS-PAGE and stained with Coomasie Brilliant Blue. Designations of mutations are as in Table 3. Asterisks denote signal of putative Gly m Bd 30 K protein. M, molecular weight marker. **b** Immunoblot analysis using polyclonal antibody against Gly m Bd 28 K protein. **c** Immunoblot analysis using polyclonal antibody against Gly m Bd 30 K protein. Gly m Bd 30 K protein. Images of full-length gel and blots are provided in additional file 2 (Figure S8)

We selected two Enrei haplotypes (E-type1 and E-type3) and seven Kariyutaka haplotypes (K-type2, K-type4, K-type7, K-type9, K-type14, K-type15, and K-type19) from the double-mutants (Table 3), and examined the composition of crude protein fractions prepared from mature seeds. The Gly m Bd 30 K protein was visually detectable in Enrei and Kariyutaka but not mutant seeds, whereas Gly m Bd 28 K was not detectable in any seeds in the SDS-PAGE analysis (Fig. 4a). The double-mutant seeds had no signal bands that were not detected in wild-type seeds (Fig. 4a). To detect the Gly m Bd 28 K and Gly m Bd 30 K proteins specifically, immunoblot analysis was conducted in double-mutant and wild-type seeds. In the immunoblot analysis, the Gly m Bd 28 K and Gly m Bd 30 K proteins were detected only in seeds of wild-type Enrei or Kariyutaka, except that Gly m Bd 28 K was also detected in the E-type3 haplotype (Fig. 4b, c). No immunoreactive band of unexpected size was detected (Additional file 2: Figure S8).

### Expression levels of the *Gly m Bd 28 K* and the *Gly m Bd 30 K* genes

**Fig. 5:**
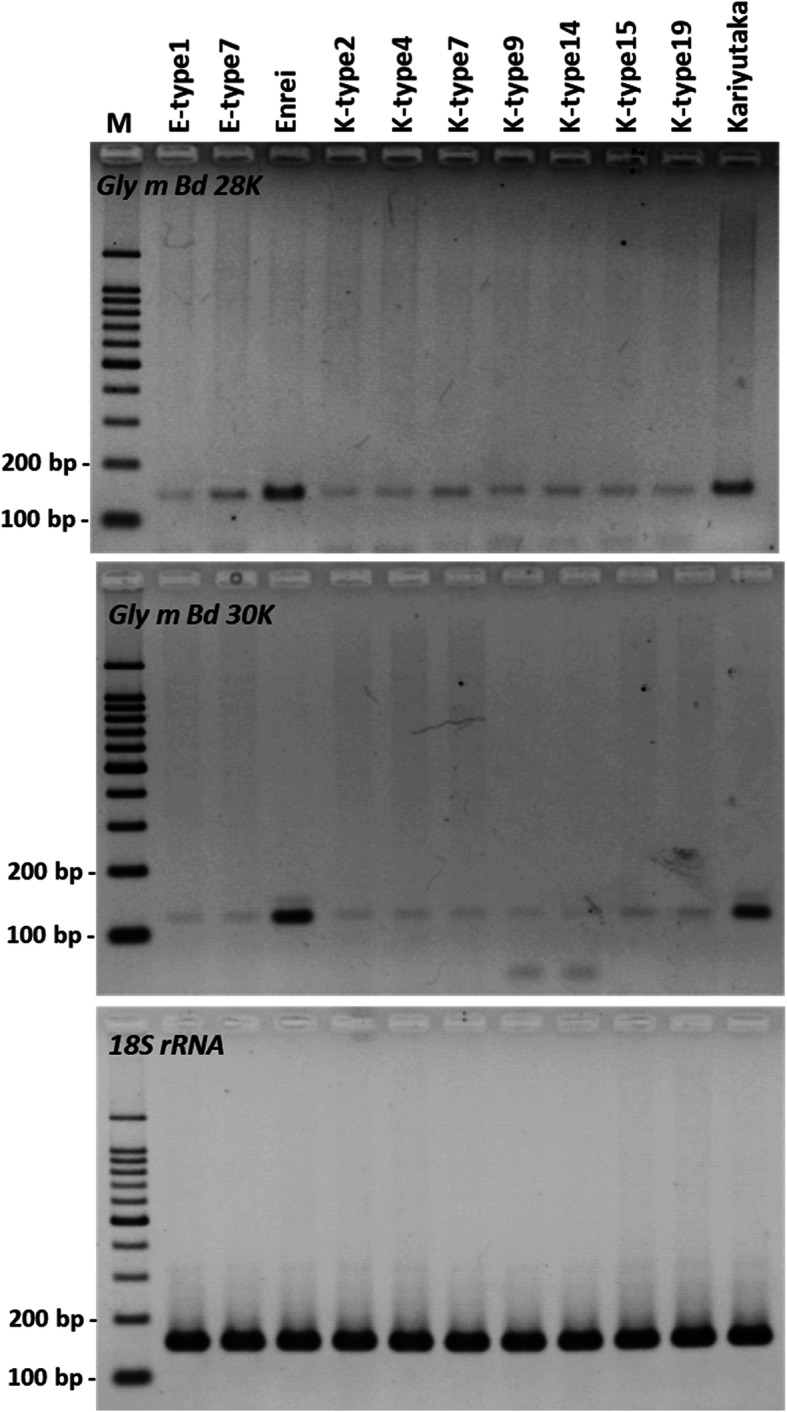
Semi-quantitative RT-PCR of the *Gly m Bd 28 K* and the *Gly m Bd 30 K* genes mature seeds of representative double-mutant T_3_ and wild-type mature seeds. The expression level of targeted loci was evaluated based on the quantity amplified for *18S rRNA* as a control endogenous gene. PCR was performed at 38 cycles for the *Gly m Bd 28 K* and at 30 cycles for the *Gly m Bd 30 K* and *18S rRNA*

To evaluate the expression levels of the *Gly m Bd 28 K* and the *Gly m Bd 30 K* genes, we extracted total RNA from the mature T_3_ seeds of two Enrei mutants (E-type1 and E-type3), seven Kariyutaka mutants (K-type2, K-type4, K-type7, K-type9, K-type14, K-type15, and K-type19), Enrei and Kariyutaka, and conducted semi-quantitative RT-PCR analysis of the region up-stream of the mutation site (Additional file 2: Figure S9). Although amplified products of the 18S ribosomal RNA (18S rRNA) were detected at similar levels in all mature seeds of the mutants, Enrei, and Kariyutaka (Fig. 5), all mutants showed lower expression levels of the *Gly m Bd 28 K* and *Gly m Bd 30 K* genes than those of wild-type (Enrei and Kariyutaka) seeds (Fig. 5).

### Whole-genome sequencing in T_2_ plants to validate the absence of foreign DNA

**Fig. 6:**
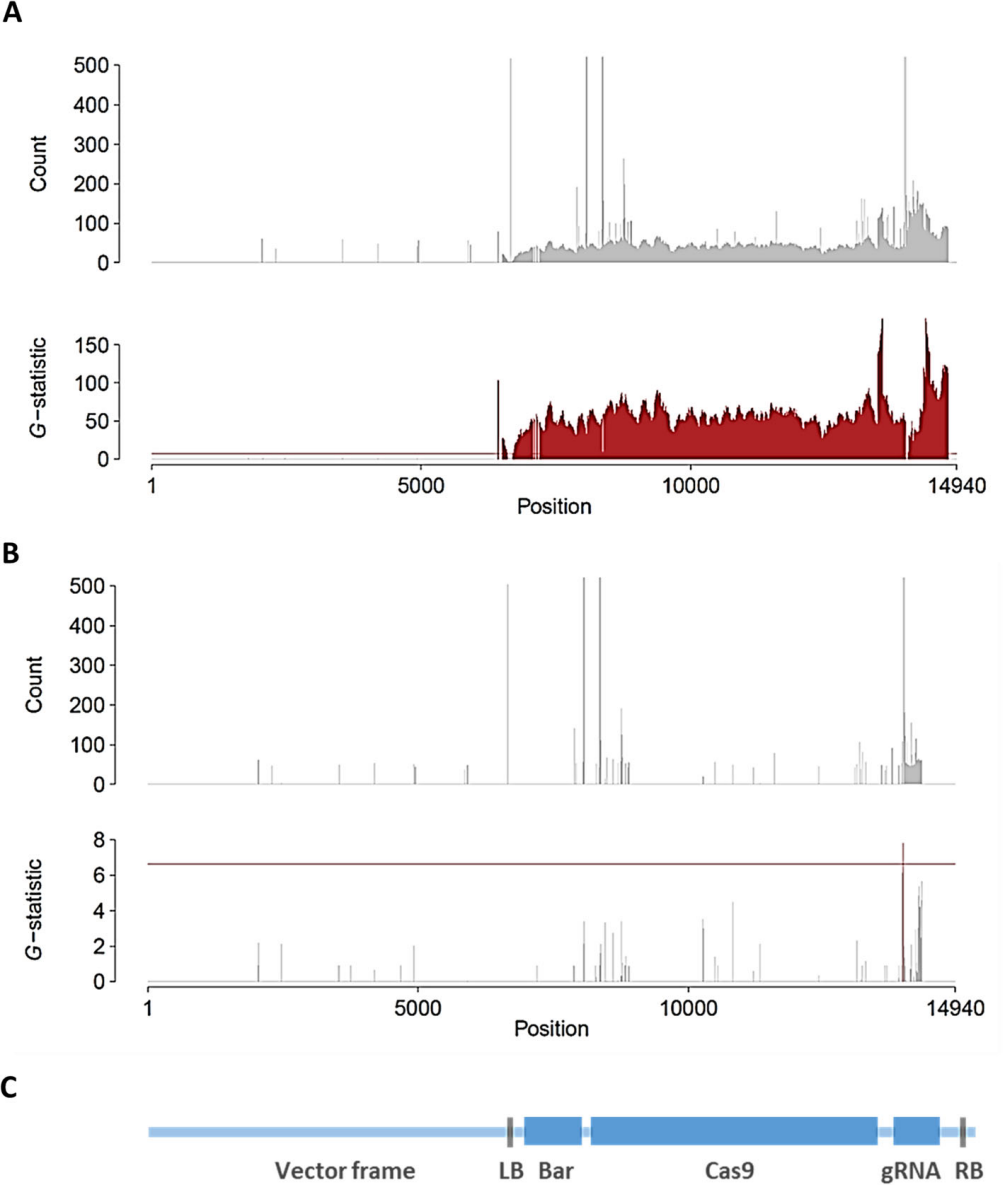
Detection of unintended remaining foreign DNA in T_2_ plants. The counts of *k*-mer and *G*-statistic are shown for (**a**) a transgenic sample K2–1-16 and (**b**) a *Cas9*-free sample K4–1-37. **c** Structure of the binary expression vector pMR284_28K_30K. The horizontal axis indicates the nucleotide positions in the vector; horizontal red line is the 1% significance level by *G*-test. *G*-statistic values exceeding the 1% level of significance are in red. The *k*-mer counts over 500 are omitted

T_2_ mutant plants K2–1-16 and K4–1-37 were selected for whole-genome sequencing analysis. These plants had homozygous mutant alleles in both loci. The K2–1-16 plant had mutant alleles with a single-nucleotide insertion in the *Gly m Bd 28 K* and a 2-nucleotide deletion in the *Gly m Bd 30 K* loci. The genome of K4–1-37 contained mutant alleles with a 6-nucleotide deletion in the *Gly m Bd 28 K* locus and a 2-nucleotide deletion in the *Gly m Bd 30 K* locus. PCR analysis detected the presence of the *Cas9* gene in the genome of K2–1-16, whereas K4–1-37 was *Cas9* free. The whole genomes of the two T_2_ plants were sequenced, and the presence of foreign DNA was examined by the *k*-mer detection method [42]. Each 20-mer identical between the plant genome and the vector was detected (Fig. 6). The genome of K2–1-16 clearly showed significant signals in a vector-wide manner (Fig. 6a, c), whereas that of K4–1-37 had no signal of foreign DNA from the vector (Fig. 6b). A significant signal found in the *G*-statistic of K4–1-37 was considered as a false positive, because it had a much lower value than that of K2–1-16.

### Morphological characteristics of double mutants

To assess the consequences of the site-directed mutagenesis in the targeted loci, we examined the morphology of the mutant plants. Because many mutant alleles in the targeted loci were detected in the Kariyutaka T_2_ generation (Fig. 3), we examined the morphological characteristics of T_2_ plant body and T_3_ seed size and shape. No difference was detected between the double-mutants and wild-type in the plant and seed morphological characteristics (Additional file 2: Figures S10, S11).

## Discussion

Gly m Bd 28 K and Gly m Bd 30 K are the major allergenic proteins in soybean seeds [6, 43]. The mutant alleles of these loci have been identified by surveying the soybean germplasm or generated by gamma-ray irradiation mutagenesis [19, 44, 45], and stacking of these mutant alleles will enable development of hypoallergenic soybean lines. In contrast, site-directed mutagenesis mediated by the CRISPR/Cas9 system enables the induction of mutations directly in the targeted loci of the desirable donor plants such as varieties and elite breeding lines. This approach dramatically shortens breeding period and saves labor. In this study, we performed simultaneous site-directed mutagenesis of both *Gly m Bd 28 K* and *Gly m Bd 30 K* loci in two Japanese soybean varieties. A total of 14 T_2_-generation seeds possessed mutant alleles of both loci and had the *Cas9* gene removed through genetic segregation (Table 3). Among all mutations, deletions were predominant and caused frame-shifts (Additional file 2: Figures S4–S6). The frame-shift mutations resulted in the deficiency in proteins recognized by the polyclonal antibodies against Gly m Bd 28 K and Gly m Bd 30 K proteins (Fig. 4). No bands of unexpected size were detected with either of these antibodies (Fig. 4). Frame-shift mutations in the targeted loci decreased the expression levels of the *Gly m Bd 28 K* and the *Gly m Bd 30 K* genes (Fig. 5). These findings suggest that the frame-shift mutations produce aberrant mRNAs from the targeted locus, which induced nonsense mRNA decay (NMD), like in a site-directed mutagenesis study conducted in *Brassica carinata* using the hairy root transformation system [46]. The lower expression level than wild-type might result in the deficiency in proteins recognized by the polyclonal antibodies against Gly m Bd 28 K and Gly m Bd 30 K proteins. On the other hand, several T_3_ seeds had mutant alleles with putative in-frame mutations (Additional file 2: Figures S6, S7). The E-type3 haplotype with a 3-nucleotide deletion in the *Gly m Bd 28 K* locus showed a strong immunoreactive band with the antibody against the Gly m Bd 28 K protein, whereas the expression level of the *Gly m Bd 28 K* gene was lower than that in Enrei (Fig. 5). In this study, the expression level of the targeted loci was examined in only mature seeds of representative mutants and wild-type. Soybean seeds accumulate Gly m Bd 28 K and Gly m Bd 30 K proteins during seed filling [47]. Therefore, an investigation of the expression level of the targeted loci in immature seeds might lead to further understanding of accumulation mechanism of mutant proteins.

At least three immunodominant epitopes in Gly m Bd 28 K and five in Gly m Bd 30 K have been identified [48–50]. In this study, gRNAs were designed against the fourth exon of *Gly m Bd 28 K* and first exon of *Gly m Bd 30 K* (Fig. 1). Immunodetection of proteins generated by the in-frame mutations in T_3_ seeds would indicate the presence proteins with preserved epitopes (Additional file 2: Figures S6, S7). Analysis of sera of soybean-allergic patients may further clarify the allergenic properties of soybean seeds generated in this study.

**Fig. 7:**
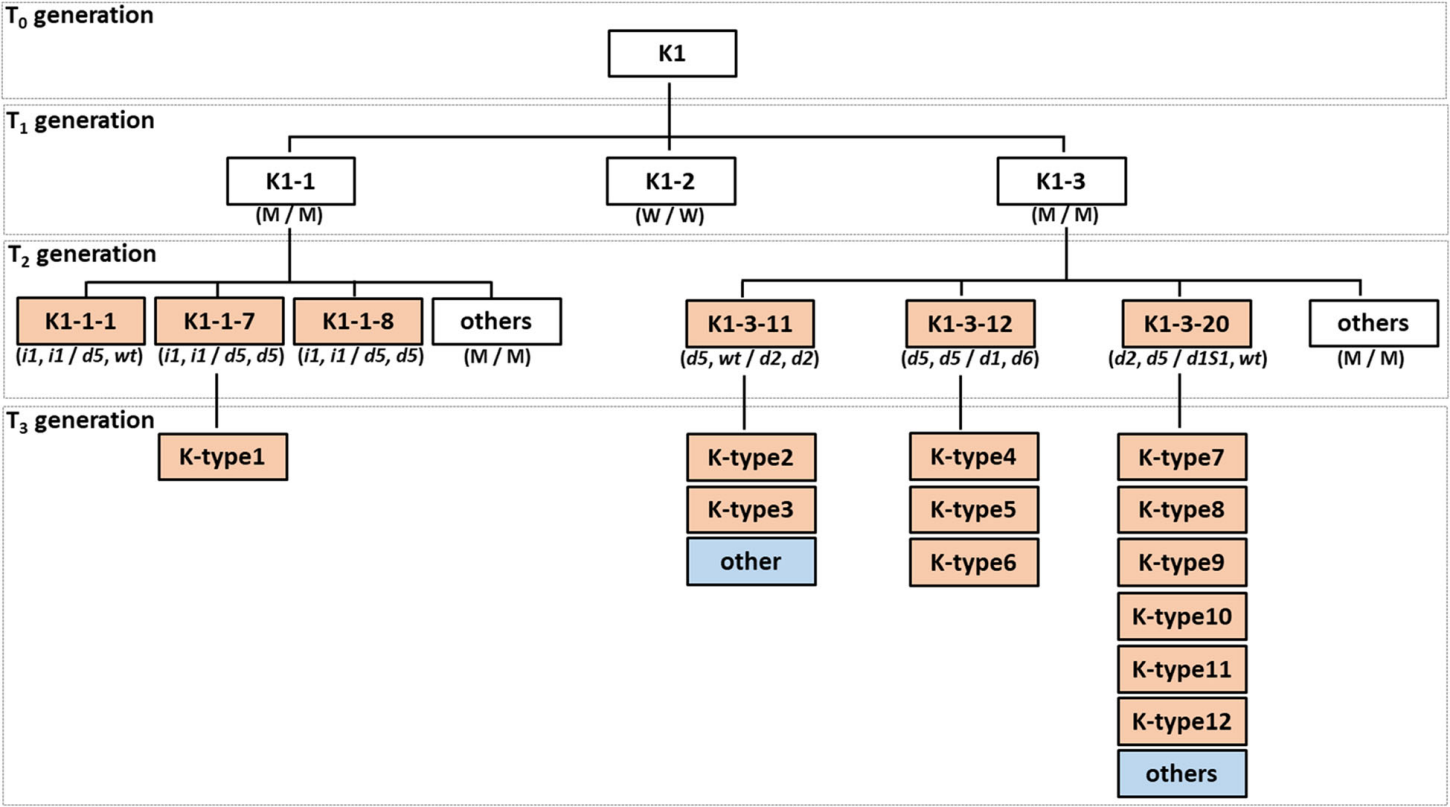
Multiple mutant alleles in the targeted loci in descendants of one T_0_ plant (K1). White boxes, transgenic; light orange boxes, *Cas9*-free double-mutants; light blue boxes, *Cas9*-free single mutants in the targeted loci. In parentheses (T_0_ and T_1_): M, mutant type; W, wild type. Designations of mutations (T_3_) are as in Table 3. In parentheses, the left description of “/” refers to genotypes of *Gly m Bd 28 K* locus and the right one to genotypes to *Gly m Bd 30 K* locus in all transgenic generations

Multiple mutant alleles were detected in the progeny of one T_0_ plant (Fig. 7). Three mutant alleles (*i1*, *d2*, and *d5*) in the *Gly m Bd 28 K* locus and five (*d1*, *d2*, *d5*, *d6*, and *d1s1*) mutant alleles in the *Gly m Bd 30 K* locus were ascertained in the *Cas9*-free T_2_ and T_3_ seeds derived from the K1 T_0_ plant (Fig. 7). These mutations appeared after the T_2_ generation, when the distribution of mutant alleles in the targeted loci was validated in the genealogy of the K1 plant and its progeny (Fig. 7). Twelve haplotypes (K-type1 to K-type12) were consequently obtained in the *Cas9*-free T_3_ seeds (Fig. 7). Previously, we showed that simultaneous site-directed mutagenesis of duplicated loci using a single gRNA resulted in heterozygous and/or chimeric mutations in the targeted loci in most of the T_1_ plants [36]. On the other hand, the mutant alleles of multiple targeted loci have been induced in early generations such as T_0_ or T_1_ plants in other studies on soybean site-directed mutagenesis by the CRISPR/Cas9 system [37, 51, 52]. This difference might be explained by different growth and maturity habits of the soybean varieties used. Kariyutaka has early flowering and a short period of vegetative growth [53]; the latter might decrease the chance of the occurrence of mutations in germ cells in the T_0_ generation, however, might produce multiple mutant alleles after the T_1_ generation. Therefore, the site-directed mutagenesis using Kariyutaka might be useful system for obtaining multiple mutant alleles in targeted genes efficiently in a limited number of transgenic soybean plants.

## Conclusion

We used *Agrobacterium*-mediated transformation and two gRNAs for simultaneous site-directed mutagenesis of two allergenic genes, *Gly m Bd 28 K* and *Gly m Bd 30 K*, in two Japanese soybean varieties. *Cas9*-free plants that had mutant alleles of the targeted loci and transgenes excluded by genetic segregation were obtained in the T_2_ or T_3_ generation. Immunoblot analysis revealed that the double-mutants did not accumulate Gly m Bd 28 K or Gly m Bd 30 K protein in mature seeds. Our results showed that simultaneous site-directed mutagenesis by the CRISPR/Cas9 system removed two major allergenic proteins in mature soybean seeds.

## Methods

### Vector construction

We constructed a gRNA expression vector (pLeg-base) which contained two gRNA expression cassettes. The frame sequence of the gRNA scaffold was derived from the vector pEn-Chimera [54]. Promoter regions of Arabidopsis U6–26 [54] and soybean U6–16g [55] were used to control gRNA and gRNA scaffold expression (Fig. 1b). The soybean allergenic genes *Gly m Bd 28 K* (Glyma.U020300.1) and *Gly m Bd 30 K* (Glyma.08G116300.1) were the targets for simultaneous site-directed mutagenesis (Fig. 1a). Two 20-nucleotide sequences (5′-CTCAGCGAACCGGATATTGG-3′ and 5′-ACCCAAGTAAAGTACCAAGG-3′) identical in each gene were used to design the gRNA sequences with the web-based CRISPR-P 2.0 (http://crispr.hzau.edu.cn/CRISPR2/). The pLeg-base vector was digested with the BbsI or BsaI restriction enzyme (NEB, Ipswich, USA). Oligonucleotides designed to match the gene-specific sequence were annealed to each other to form the gRNA seed sequence, which was ligated into pLeg-base. The CRISPR/Cas9 expression plasmid (pMR284_28K_30K) was constructed by inserting the gRNA expression cassettes of pLeg-base into a Cas9-binary vector (pMR284) harboring *Cas9* and glufosinate resistance gene (*Bar*) expression cassettes using LR Clonase (Thermo Fisher Scientific, Waltham, USA).

### Soybean transformation

The Japanese soybean varieties Enrei (JP 28862) and Kariyutaka (JP 86520) were obtained from Genebank, National Agriculture and Food Research Organization (https://www.gene.affrc.go.jp/index_en.php). *Agrobacterium*-mediated transformation was performed as described in [28], except that the concentration of glufosinate for selection of transformed cells was decreased from 6 mg/L to 4 mg/L for Enrei. *Agrobacterium tumefaciens* EHA105 harboring the plasmid pMR284_28K_30K was used. Transgenic plants were grown in commercial soil (Katakura Chikkarin Co., Tokyo, Japan) at 25 °C in an isolated greenhouse for transgenic plants.

### Extraction of genomic DNA and detection of mutations in targeted loci by CAPS analysis

To extract leaf genomic DNA, leaf pieces (approximately 5 mm × 5 mm) were homogenized in 200 μL of extraction buffer [2% CTAB (hexadecyltrimethyl-ammonium bromide), 100 mM Tris-HCl (pH 8.0), 20 mM EDTA (pH 8.0), 1.4 M NaCl, and 0.07% 2-mercaptethanol] in a BioMasher II tube (Nippi, Tokyo, Japan). To extract genomic DNA from mature seeds, a part of cotyledon was powdered and approximately 5 mg of powder was stirred in extraction buffer [10 mM Tris-HCl (pH 8.0), 5 mM EDTA, 0.5% SDS, 0.5% NP40, 0.5% Tween 20, and 80 mg/L proteinase-K (Wako, Osaka, Japan)]. The mixture was incubated at 50 °C for 1 h. DNA extracts were deproteinized with a mixture of phenol, chloroform, and isoamyl alcohol (25:24:1). DNA was precipitated from the supernatant with 2-propanol. The targeted regions in the *Gly m Bd 28 K* and *Gly m Bd 30 K* loci were amplified by PCR with specific primers (Additional file 1: Table S3). The PCR was performed under the following conditions: 30 cycles of 94 °C for 30 s, 54 °C (the *Gly m Bd 28 K*) or 60 °C (the *Gly m Bd 30 K*) for 30 s and 72 °C for 60 s. The amplified products were digested with the DdeI and BsaJI restriction enzymes (NEB), respectively, and separated by electrophoresis in 2.0% agarose gels. The DNA fragments of expected digested-pattern derived from the targeted region carrying mutations and those with no mutations were considered as the mutant type and wild type, respectively. DNA fragments of unexpected size were also regarded as mutant type.

### DNA sequencing

The targeted and flanking regions of the *Gly m Bd 28 K* and *Gly m Bd 30 K* loci were amplified with specific primers (Additional file 1: Table S3). The amplified products were cloned into the pGEM-T-Easy vector (Promega, Madison, USA) and sequenced with the Big Dye terminator cycle method using an ABI3100 or ABI3130 Genetic Analyzer (Thermo Fisher Scientific). DNA sequencing analysis was performed by the Instrumental Analysis Division, Graduate School of Agriculture, Hokkaido University.

### Selection of *Cas9*-free plants

To confirm the integration of the Cas9 and gRNA expression module in T_1_–T_3_ generations, PCR analysis was performed using primers specific for the *Cas9* gene (Additional file 1: Table S3). PCR was also performed to simultaneously amplify endogenous Glyma.01G214600 as a positive control. The PCR was performed under the following conditions: 30 cycles of 94 °C for 30 s, 54 °C for 30 s and 72 °C for 30 s. The existence of the *Cas9* gene were identified by the existence of products amplified by the PCR.

### Protein analyses in mature seeds

Soy meal was collected from mature seeds. The extraction of crude protein and protein separation were performed as described in [28]. Proteins were separated by SDS-PAGE in a precast 5–12% gradient gel (ATTO, Tokyo, Japan) and transferred onto a PVDF membrane (Hybond-P; GE Healthcare, Little Chalfont, UK). Membranes were blocked with 5% skim milk (Wako) overnight at 4 °C. Recombinant Gly m Bd 30 k was prepared using the baculovirus expression system as described in [5]. Using the pET52 vector (Merck-Millipore, Burlington, USA), His_10_-tagged Gly m Bd 28 K was expressed in *Escherichia coli* BL21(DE3). After sonication and centrifugation, Gly m Bd 28 K–containing pellets were dissolved in phosphate-buffered saline containing 8 M urea, and Gly m Bd 28 K was purified using a HisTrapFF crude column (GE Healthcare). Antisera were raised in rabbits against the recombinant proteins as described in [56]. Immunoreactive bands were detected with the antisera and the ECL Plus Western Blotting system (GE Healthcare).

### Expression analysis by semi-quantitative RT-PCR

Total RNA was extracted from mature seeds of mutants, Enrei, and Kariyutaka by the LiCl precipitation procedure [28]. Semi-quantitative RT-PCR was conducted in a 20-μL volume using 30 or 38 cycles of 94 °C for 30 s, 57 °C for 30 s, and 72 °C for 10 s. The transcript level of the *Gly m Bd 28 K* and the *Gly m Bd 30 K* gene was evaluated relative to that of the *18S rRNA* gene (XR_003264275).

### Genome sequencing

Total DNA was isolated from fresh leaves (1.0–2.0 g) of wild-type and T_2_ plants as described in [41]. Genomic DNA libraries were constructed using a TruSeq DNA PCR-Free Library Preparation Kit (Illumina, San Diego, USA).Whole-genome sequencing was conducted on an Illumina HiSeq X platform to obtain 151-nt paired-end reads. Approximately 50× coverage data were obtained for each sample. Unintended remaining foreign DNA was detected as described in [42].

## Supplementary Information

**Additional file 1: Table S1.** Induction of mutations in the targeted loci and the integration of the *Cas9* gene in representative T_1_ plants from the transformation of Enrei. **Table S2.** Induction of mutations in the targeted loci and the integration of the *Cas9* gene in representative T_1_ plants from the transformation of Kariyutaka. **Table S3.** Primer sequences used for vector construction, confirmation of transgenes, and CAPS, semi-quantitative RT-PCR, and sequencing analyses.

**Additional file 2: Figure S1.** Confirmation of mutagenesis of targeted loci in representative Kariuytaka-T_1_ plants by CAPS analysis. **Figure S2.** Detection of mutations in the *Gly m Bd 28K* and *Gly m Bd 30K* loci in representative Enrei-T_2_ seeds by CAPS analysis. **Figure S3.** Detection of mutations in the *Gly m Bd 28K* and *Gly m Bd 30K* loci in representative Kariyutka-T_2_ seeds by CAPS analysis. **Figure S4.** Detection of the integration of the *Cas9* gene in representative T_2_ seeds by PCR analysis. **Figure S5.** Mutational spectra of the targeted loci in double-mutant T_3_ seeds. **Figure S6.** Alignment of predicted amino acid sequences of the *Gly m Bd 28K* locus in double mutants. **Figure S7.** Alignment of predicted amino acid sequences of the *Gly m Bd 30K* locus in double mutants. **Figure S8.** Full-length gel electrophoresis and immunoblot of the crude protein of representative double-mutant T_3_ and wild-type mature seeds. **Figure S9.** Primer sites used for semi-quantitative RT-PCR analysis of the *Gly m Bd 30K* and the *Gly m Bd 30K* loci. **Figure S10.** Morphological characteristics of representative double-mutant (T_2_) and control Kariyutaka plants. **Figure S11.** Morphological characteristics of representative double-mutant (T_3_) and control Kariyutaka seeds.

## Data Availability

The datasets supporting this study are included within the manuscript and its additional files. The vector developed in this study is available from the corresponding author on reasonable request. The datasets of genomic sequence in Kariyutaka and mutants (K2–1-16 and K4–1-37) have been deposited in the DDBJ Sequence Read Archive under the BioProject Accession PRJDB10633.

## Abbreviations

PVDF: Polyvinylidene difluoride
SDS-PAGE: Sodium dodecyl sulfate-polyacrylamide gel electrophoresis

## References

[1] Liu K (1997). Soybean as functional foods and ingredients.

[2] Utsumi S, Damodaran S, Kinsella JE (1984). Heat-induced interactions between soybean proteins - preferential association of 11S basic subunits and beta-subunits of 7S. J Agric Food Chem.

[3] Nagano T, Mori H, Nishinari K (1994). Effect of heating and cooling on the gelation kinetics of 7S globulin from soybeans. J Agric Food Chem.

[4] Heppell LM, Sissons JW, Pedersen HE (1987). A comparison of the antigenicity of soya-bean-based infant formulas. Br J Nutr.

[5] Maruyama N, Sato S, Cabanos C, Tanaka A, Ito K, Ebisawa M (2018). Gly m 5/Gly m 8 fusion component as a potential novel candidate molecular for diagnosing soya bean allergy in Japanese children. Clin Exp Allergy.

[6] Ogawa T, Tsuji H, Bando N, Kitamura K, Zhu YL, Hirano H (1993). Identification of the soybean allergenic protein, Gly-m Bd 30k, with the soybean seed 34-kDa oil-body-associated protein. Biosci Biotechnol Biochem.

[7] Tsuji H, Bando N, Hiemori M, Yamanishi R, Kimoto M, Nishikawa K (1997). Purification and characterization of soybean allergen Gly m Bd 28K. Biosci Biotechnol Biochem.

[8] Gonzalez R, Polo F, Zapatero L, Caravaca F, Carreira J (1992). Purification and characterization of major inhalant allergens from soybean hulls. Clin Exp Allergy.

[9] Codina R, Lockey RF, Fernandez-Caldas E, Rama R (1997). Purification and characterization of a soybean hull allergen responsible for the Barcelona asthma outbreaks. 2. Purification and sequencing of the Gly m 2 allergen. Clin Exp Allergy.

[10] Rihs HP, Chen ZP, Rueff F, Petersen A, Rozynek P, Heimann H (1999). IgE binding of the recombinant allergen soybean profilin (rGly m 3) is mediated by conformational epitopes. J Allergy Clinic Immun.

[11] Kleine-Tebbe J, Wangorsch A, Vogel L, Crowell DN, Haustein UF, Vieths S (2002). Severe oral allergy syndrome and anaphylactic reactions caused by a bet v 1-related PR-10 protein in soybean, SAM22. J Allergy Clinic Immun.

[12] Ogawa T, Samoto M, Takahashi K (2000). Soybean allergens and hypoallergenic soybean products. J Nutr Sci Vitaminol.

[13] Thanh VH, Shibasaki K (1976). Major proteins of soybean seeds - straightforward fractionation and their characterization. J Agric Food Chem.

[14] Iwabuchi S, Yamauchi F (1987). Electrophoretic analysis of whey proteins present in soybean globulin fractions. J Agric Food Chem.

[15] Samoto M, Maebuchi M, Miyazaki C, Kugitani H, Kohno M, Hirotsuka M (2007). Abundant proteins associated with lecithin in soy protein isolate. Food Chem.

[16] Samoto M, Akasaka T, Mori H, Manabe M, Ookura T, Kawamura Y (1994). Simple and efficient procedure for removing the 34kDa allergenic soybean protein, *Gly m* I, from defatted soy milk. Biosci Biotechnol Biochem.

[17] Mori T, Utsumi S, Inaba H, Kitamura K, Harada K (1981). Differences in composition of glycinin among soybean cultivars. J Agric Food Chem.

[18] Kitamura K, Davies CS, Nielsen NC (1984). Inheritance of alleles for Cgy_1_ and Gy_4_ storage protein genes in soybean. Theor Appl Genet.

[19] Takahashi K, Banba H, Kikuchi A, Ito M, Nakamura S (1994). An induced mutant line lacking the α-subunit of β-conglycinin in soybean [*Glycine max* (L.) Merrill]. Breed Sci.

[20] Hajika M, Takahashi M, Sakai SJ, Igita K (1996). A new genotype of 7 S globulin (beta-conglycinin) detected in wild soybean (*Glycine soja* Sieb et Zucc). Breed Sci.

[21] Tsubokura Y, Hajika M, Kanamori H, Xia ZJ, Watanabe S, Kaga A (2012). The β-conglycinin deficiency in wild soybean is associated with the tail-to-tail inverted repeat of the α-subunit genes. Plant Mol Biol.

[22] Hajika M, Takahashi M, Sakai S, Matsunaga R (1998). Dominant inheritance of a trait lacking β-conglycinin detected in a wild soybean line. Breed Sci.

[23] Takahashi K, Mizuno Y, Yumoto S, Kitamura K, Nakamura S (1996). Inheritance of the α-subunit deficiency of β-conglycinin in soybean (*Glycine max* L MERRILL) line induced by gamma-ray irradiation. Breed Sci.

[24] Manjaya JG, Suseelan KN, Gopalakrishna T, Pawar SE, Bapat VA (2007). Radiation induced variability of seed storage proteins in soybean *Glycine max* (L.) Merrill. Food Chem.

[25] Schmidt MA, Hymowitz T, Herman EM (2015). Breeding and characterization of soybean triple null; a stack of recessive alleles of Kunitz trypsin inhibitor, soybean agglutinin, and P34 allergen nulls. Plant Breed.

[26] Herman EM, Helm RM, Jung R, Kinney AJ (2003). Genetic modification removes an immunodominant allergen from soybean. Plant Physiol.

[27] Nishizawa K, Takagi K, Teraishi M, Kita A, Ishimoto M (2010). Application of somatic embryos to rapid and reliable analysis of soybean seed components by RNA interference-mediated gene silencing. Plant Biotechnol.

[28] Yamada T, Mori Y, Yasue K, Maruyama N, Kitamura K, Abe J (2014). Knockdown of the 7S globulin subunits shifts distribution of nitrogen sources to the residual protein fraction in transgenic soybean seeds. Plant Cell Rep.

[29] Cermak T, Doyle EL, Christian M, Wang L, Zhang Y, Schmidt C (2011). Efficient design and assembly of custom TALEN and other TAL effector-based constructs for DNA targeting. Nucleic Acids Res.

[30] Li JF, Norville JE, Aach J, McCormack M, Zhang DD, Bush J (2013). Multiplex and homologous recombination-mediated genome editing in *Arabidopsis* and *Nicotiana benthamian*a using guide RNA and Cas9. Nat Biotechnol.

[31] Nekrasov V, Staskawicz B, Weigel D, Jones JDG, Kamoun S (2013). Targeted mutagenesis in the model plant *Nicotiana benthamiana* using Cas9 RNA-guided endonuclease. Nat Biotechnol.

[32] Shan QW, Wang YP, Li J, Zhang Y, Chen KL, Liang Z (2013). Targeted genome modification of crop plants using a CRISPR-Cas system. Nat Biotechnol.

[33] Li ZS, Liu ZB, Xing AQ, Moon BP, Koellhoffer JP, Huang LX (2015). Cas9-guide RNA directed genome editing in soybean. Plant Physiol.

[34] Cai Y, Chen L, Liu X, Guo C, Sun S, Wu C (2018). CRISPR/Cas9-mediated targeted mutagenesis of *GmFT2a* delays flowering time in soybean. Plant Biotechnol J.

[35] Curtin SJ, Xiong Y, Michno J-M, Campbell BW, Stec AO, Čermák T (2018). CRISPR/Cas9 and TALENs generate heritable mutations for genes involved in small RNA processing of *Glycine max* and *Medicago truncatula*. Plant Biotechnol J.

[36] Kanazashi Y, Hirose A, Takahashi I, Mikami M, Endo M, Hirose S (2018). Simultaneous site-directed mutagenesis of duplicated loci in soybean using a single guide RNA. Plant Cell Rep.

[37] Do PT, Nguyen CX, Bui HT, Tran LTN, Stacey G, Gillman JD (2019). Demonstration of highly efficient dual gRNA CRISPR/Cas9 editing of the homeologous *GmFAD2-1A* and *GmFAD2-1B* genes to yield a high oleic, low linoleic and α-linolenic acid phenotype in soybean. BMC Plant Biol.

[38] Nakamura T, Utsumi S, Kitamura K, Harada K, Mori T (1984). Cultivar differences in gelling characteristics of soybean glycinin. J Agric Food Chem.

[39] Tezuka M, Taira H, Igarashi Y, Yagasaki K, Ono T (2000). Properties of tofus and soy milk prepared from soybeans having different subunits of glycinin. J Agric Food Chem.

[40] Maruyama N, Park K, Motoyama S, Choi S-K, Yagasaki K, Ishimoto M (2004). Structure–physicochemical function relationships of soybean glycinin at subunit levels assessed by using mutant lines. J Agric Food Chem.

[41] Yamada T, Watanabe S, Arai M, Harada K, Kitamura K (2010). Cotyledonary node pre-wounding with a micro-brush increased frequency of agrobacterium-mediated transformation in soybean. Plant Biotechnol.

[42] Itoh T, Onuki R, Tsuda M, Oshima M, Endo M, Sakai H (2020). Foreign DNA detection by high-throughput sequencing to regulate genome-editing agricultural products. Sci Rep.

[43] Ogawa T, Bando N, Tsuji H, Okajima H, Nishikawa K, Sasaoka K (1991). Investigation of the IgE-binding proteins in soybeans by immunoblotting with the sera of the soybean-sensitive patients with atopic-dermatitis. J Nutr Sci Vitaminol.

[44] Samoto M, Fukuda Y, Takahashi K, Tabuchi K, Hiemori M, Tsuji H (1997). Substantially complete removal of three major allergenic soybean proteins (Gly m Bd 30K, Gly m Bd 28K, and the α-subunit of conglycinin) from soy protein by using a mutant soybean, Tohoku 124. Biosci Biotechnol Biochem.

[45] Joseph LM, Hymowitz T, Schmidt MA, Herman EM (2006). Evaluation of *Glycine* germplasm for nulls of the immunodominant allergen P34/Gly m Bd 30k. Crop Sci.

[46] Kirchner TW, Niehaus M, Debener T, Schenk MK, Herde M (2017). Efficient generation of mutations mediated by CRISPR/Cas9 in the hairy root transformation system of *Brassica carinata*. PLoS One.

[47] Wu YM, Guan RX, Liu ZX, Li RZ, Chang RZ, Qiu LJ (2012). Synthesis and degradation of the major allergens in developing and germinating soybean seed. J Integr Plant Biol.

[48] Helm RM, Cockrell G, Herman E, Burks AW, Sampson HA, Bannon GA (1998). Cellular and molecular characterization of a major soybean allergen. Int Arch Allergy Immunol.

[49] Helm RM, Cockrell G, Connaughton C, West CM, Herman E, Sampson HA (2000). Mutational analysis of the IgE-binding epitopes of P34/Gly m Bd 30K. J Allergy Clin Immunol.

[50] Xi J, Yan HL (2016). Epitope mapping and identification of amino acids critical for mouse IgG-binding to linear epitopes on Gly m Bd 28K. Biosci Biotechnol Biochem.

[51] Cai YP, Wang LW, Chen L, Wu TT, Liu LP, Sun S (2020). Mutagenesis of *GmFT2a* and *GmFT5a* mediated by CRISPR/Cas9 contributes for expanding the regional adaptability of soybean. Plant Biotechnol J.

[52] Bao AL, Chen HF, Chen LM, Chen SL, Hao QN, Guo W (2019). CRISPR/Cas9-mediated targeted mutagenesis of *GmSPL9* genes alters plant architecture in soybean. BMC Plant Biol.

[53] Tsubokura Y, Watanabe S, Xia Z, Kanamori H, Yamagata H, Kaga A (2014). Natural variation in the genes responsible for maturity loci *E1*, *E2*, *E3* and *E4* in soybean. Ann Bot.

[54] Fauser F, Schiml S, Puchta H (2014). Both CRISPR/Cas-based nucleases and nickases can be used efficiently for genome engineering in Arabidopsis thaliana. Plant J.

[55] Du HY, Zeng XR, Zhao M, Cui XP, Wang Q, Yang H (2016). Efficient targeted mutagenesis in soybean by TALENs and CRISPR/Cas9. J Biotechnol.

[56] Nishizawa K, Maruyama N, Satoh R, Fuchikami Y, Higasa T, Utsumi SA (2003). C-terminal sequence of soybean β-conglycinin α' subunit acts as a vacuolar sorting determinant in seed cells. Plant J.

