# Supplementary material for: Simultaneous induction of mutant alleles of two allergenic genes in soybean by using site-directed mutagenesis
Source: BMC Plant Biol. 2020 Nov 11;20:513. doi: 10.1186/s12870-020-02708-6 (PMC7656749; doi:10.1186/s12870-020-02708-6)
Supplement: Supplementary file 1 — Additional file 1: Table S1. Induction of mutations in the targeted loci and the integration of the Cas9 gene in representative T1 plants from the transformation of Enrei. Table S2. Induction of mutations in the targeted loci and the integration of the Cas9 gene in representative T1 plants from the transformation of Kariyutaka. Table S3. Primer sequences used for vector construction, confirmation of transgenes, and CAPS, semi-quantitative RT-PCR, and sequencing analyses. [file 12870_2020_2708_MOESM1_ESM.docx]

**Table S1.** Induction of mutations in the targeted loci and the integration of the *Cas9* gene in representative T_1_ plants from the transformation of Enrei

| T_1_ plant  number | CAPS analysis | | | |  | | | PCR analysis | |
| --- | --- | --- | --- | --- | --- | --- | --- | --- | --- |
|  | *Gly m Bd 28K* | | *Gly m Bd 30K* | |  |  |  | *Cas9* | |
|  |  | |  | |  | | |  | |
| E1-1 | M | | M | |  | | | D | |
|  |  | |  | |  | | |  | |
| E1-2 | M | | M | |  | | | D | |
|  |  | |  | |  | | |  | |
| E1-3 | M | | W | |  | | | D | |
|  |  | |  | |  | | |  | |
| E1-4 | W | | M | |  | | | ND | |
|  |  | |  | |  | | |  | |
| E1-5 | M | | M | |  | | | D | |
|  |  | |  | |  | | |  | |
| E1-6 | M | | W | |  | | | D | |
|  |  | |  | |  | | |  | |
| E1-7 | M | | M | |  | | | D | |
|  |  | |  | |  | | |  | |
| E1-8 | W | | M | |  | | | ND | |
|  |  | |  | |  | | |  | |
| E1-9 | W | | M | |  | | | ND | |
|  |  | |  | |  | | |  | |
| E1-10 | W | | W | |  | | | ND | |
|  |  | |  | |  | | |  | |
| E2-1 | M | | W | |  | | | D | |
|  |  | |  | |  | | |  | |
| E2-2 | M | | W | |  | | | D | |
|  |  | |  | |  | | |  | |
| E2-3 | M | | W | |  | | | D | |
|  |  | |  | |  | | |  | |
| E2-4 | W | | W | |  | | | ND | |
|  |  | |  | |  | | |  | |
| E2-5 | M | | W | |  | | | D | |
|  |  | |  | |  | | |  | |
| E2-6 | M | | W | |  | | | D | |
|  |  | |  | |  | | |  | |
| E2-7 | M | | W | |  | | | D | |
|  |  | |  | |  | | |  | |
| E2-8 | W | | W | |  | | | ND | |
|  |  | |  | |  | | |  | |
| E2-9 | M | | W | |  | | | D | |
|  |  | |  | |  | | |  | |
| E2-10 | M | | W | |  | | | D | |
|  |  |  |  |  | |  |  | |  |

Induction of mutation in the targeted loci and integration of the *Cas9* gene were evaluated by CAPS and PCR analyses, respectively. M, mutant type; W, wild type; D, detected; ND, not detected

**Table S2.** Induction of mutations in the targeted loci and the integration of the *Cas9* gene in representative T_1_ plants from the transformation of Kariyutaka

| T_1_ plant  number | CAPS analysis | | | |  | | | PCR analysis | |
| --- | --- | --- | --- | --- | --- | --- | --- | --- | --- |
|  | *Gly m Bd 28K* | | *Gly m Bd 30K* | |  |  |  | *Cas9* | |
|  |  | |  | |  | | |  | |
| K1-1 | M | | M | |  | | | D | |
|  |  | |  | |  | | |  | |
| K1-2 | W | | M | |  | | | ND | |
|  |  | |  | |  | | |  | |
| K1-3 | M | | M | |  | | | D | |
|  |  | |  | |  | | |  | |
| K2-1 | M | | W | |  | | | D | |
|  |  | |  | |  | | |  | |
| K2-2 | M | | M | |  | | | D | |
|  |  | |  | |  | | |  | |
| K2-3 | M | | M | |  | | | D | |
|  |  | |  | |  | | |  | |
| K2-4 | W | | W | |  | | | ND | |
|  |  | |  | |  | | |  | |
| K3-1 | W | | W | |  | | | D | |
|  |  | |  | |  | | |  | |
| K3-2 | W | | W | |  | | | D | |
|  |  | |  | |  | | |  | |
| K3-3 | W | | W | |  | | | D | |
|  |  | |  | |  | | |  | |
| K4-1 | M | | M | |  | | | D | |
|  |  | |  | |  | | |  | |
| K4-2 | W | | M | |  | | | D | |
|  |  | |  | |  | | |  | |
| K5-1 | M | | M | |  | | | D | |
|  |  | |  | |  | | |  | |
| K5-2 | M | | M | |  | | | D | |
|  |  | |  | |  | | |  | |
| K6-1 | W | | W | |  | | | ND | |
|  |  | |  | |  | | |  | |
| K6-2 | M | | M | |  | | | D | |
|  |  | |  | |  | | |  | |
| K6-3 | M | | M | |  | | | D | |
|  |  | |  | |  | | |  | |
| K6-4 | W | | W | |  | | | ND | |
|  |  | |  | |  | | |  | |
| K7-1 | W | | W | |  | | | ND | |
|  |  | |  | |  | | |  | |
| K8-1 | W | | M | |  | | | D | |
|  |  | |  | |  | | |  | |
| K9-1 | W | | M | |  | | | D | |
|  |  | |  | |  | | |  | |
| K10-1 | W | | M | |  | | | D | |
|  |  | |  | |  | | |  | |
| K10-2 | W | | M | |  | | | D | |
|  |  | |  | |  | | |  | |
| K10-3 | W | | M | |  | | | D | |
|  |  | |  | |  | | |  | |
| K10-4 | W | | M | |  | | | D | |
|  |  |  |  |  | |  |  | |  |

Induction of mutation in the targeted loci and integration of the *Cas9* gene were evaluated by CAPS and PCR analyses, respectively. M, mutant type; W, wild type; D, detected; ND, not detected

| **Table S3.** Primer sequences used for vector construction, confirming of transgenes, CAPS, and sequencing analyses. | | |
| --- | --- | --- |
| Primer name | Primer sequence (5'-3') | Use of amplicon |
| 28K-targete_Fw | GATTCCAATATCCGGTTCGCTGAG | Vector construction |
| 28K-targete_Rv | AAACCTCAGCGAACCGGATATTGG | Vector construction |
| 30K-targete_Fw | ATTGACCCAAGTAAAGTACCAAGG | Vector construction |
| 30K-targete_Rv | AAACCCTTGGTACTTTACTTGGGT | Vector construction |
| AtU6-pro_Fw | TGTTTATCAGCTTACATTTTCTTGAACCGTAGCT | Vector construction and sequencing analyses |
| GmU6-16g-pro_Fw | AAAGAAATGACAGGGCTACAAAAG | Vector construction and sequencing analyses |
| gRNA-scaffold_Rv | TAATGCCAACTTTGTACAAGAAAGCTGGGTCTAGA | Vector construction and sequencing analyses |
| 28K-PCR_Fw | CACTTTACCAGGATTACCAAC | CAPS and sequencing analyses |
| 28K-PCR_Rv | AAAGAAATGACAGGGCTACAAAAG | CAPS and sequencing analyses |
| 28K-PCR_FwE | CTACATGCACGACACGTGTCTC | Gene expression analysis |
| 28K-PCR_RvE | TCCATGGCAAAGAACAAAGAGC | Gene expression analysis |
| 30K-PCR_Fw1 | GCAAGCTCCCAAGGATGTG | CAPS and sequencing analyses |
| 30K-PCR_Fw2 | ACCATCCACCTGCATCATGG | Sequencing analysis |
| 30K-PCR_Rv | ACGCCCAACCGCTTCCTAT | CAPS and sequencing analyses |
| 30K-PCR_FwE | GTGAGCATGGACGTGTCTACC | Gene expression analysis |
| 30K-PCR_RvE | CGATGAGAATGGGGTGATTT | Gene expression analysis |
| Cas9_Fw2 | ATCCACGATGATTCTCTCACCT | Confirming of transgene(s) |
| Cas9_Rv2 | AGAATGACTGTGGCACGATATG | Confirming of transgene(s) |
| GmSGR1_F1*^a^* | GGTACTACTGTTTCATCTTCCCTC | Endogenous control for PCR analysis |
| GmSGR1_R4*^a^* | CCAAGCACCCATCTAATTGGTC | Endogenous control for PCR analysis |
| Gm18SrRNA_Fw*^b^* | TGATTAACAGGGACAGTCGG | Gene expression analysis |
| Gm18SrRNA_Rv*^b^* | ACGGTATCTGATCGTCTTCG | Gene expression analysis |

*^a^*Glyma.01G214600 was used for the endogenous control gene in the PCR analysis.

*^b^*Soybean 18S ribosomal RNA (X02623) was used as the endogenous control in the gene expression analysis.
