# Supplementary material for: Simultaneous induction of mutant alleles of two allergenic genes in soybean by using site-directed mutagenesis
Source: BMC Plant Biol. 2020 Nov 11;20:513. doi: 10.1186/s12870-020-02708-6 (PMC7656749; doi:10.1186/s12870-020-02708-6)
Supplement: Supplementary file 2 — Additional file 2: Figure S1. Confirmation of mutagenesis of targeted loci in representative Kariuytaka-T1 plants by CAPS analysis. Figure S2. Detection of mutations in the Gly m Bd 28K and Gly m Bd 30K loci in representative Enrei-T2 seeds by CAPS analysis. Figure S3. Detection of mutations in the Gly m Bd 28K and Gly m Bd 30K loci in representative Kariyutka-T2 seeds by CAPS analysis. Figure S4. Detection of the integration of the Cas9 gene in representative T2 seeds by PCR analysis. Figure S5. Mutational spectra of the targeted loci in double-mutant T3 seeds. Figure S6. Alignment of predicted amino acid sequences of the Gly m Bd 28K locus in double mutants. Figure S7. Alignment of predicted amino acid sequences of the Gly m Bd 30K locus in double mutants. Figure S8. Full-length gel electrophoresis and immunoblot of the crude protein of representative double-mutant T3 and wild-type mature seeds. Figure S9. Primer sites used for semi-quantitative RT-PCR analysis of the Gly m Bd 30K and the Gly m Bd 30K loci. Figure S10. Morphological characteristics of representative double-mutant (T2) and control Kariyutaka plants. Figure S11. Morphological characteristics of representative double-mutant (T3) and control Kariyutaka seeds. [file 12870_2020_2708_MOESM2_ESM.docx]

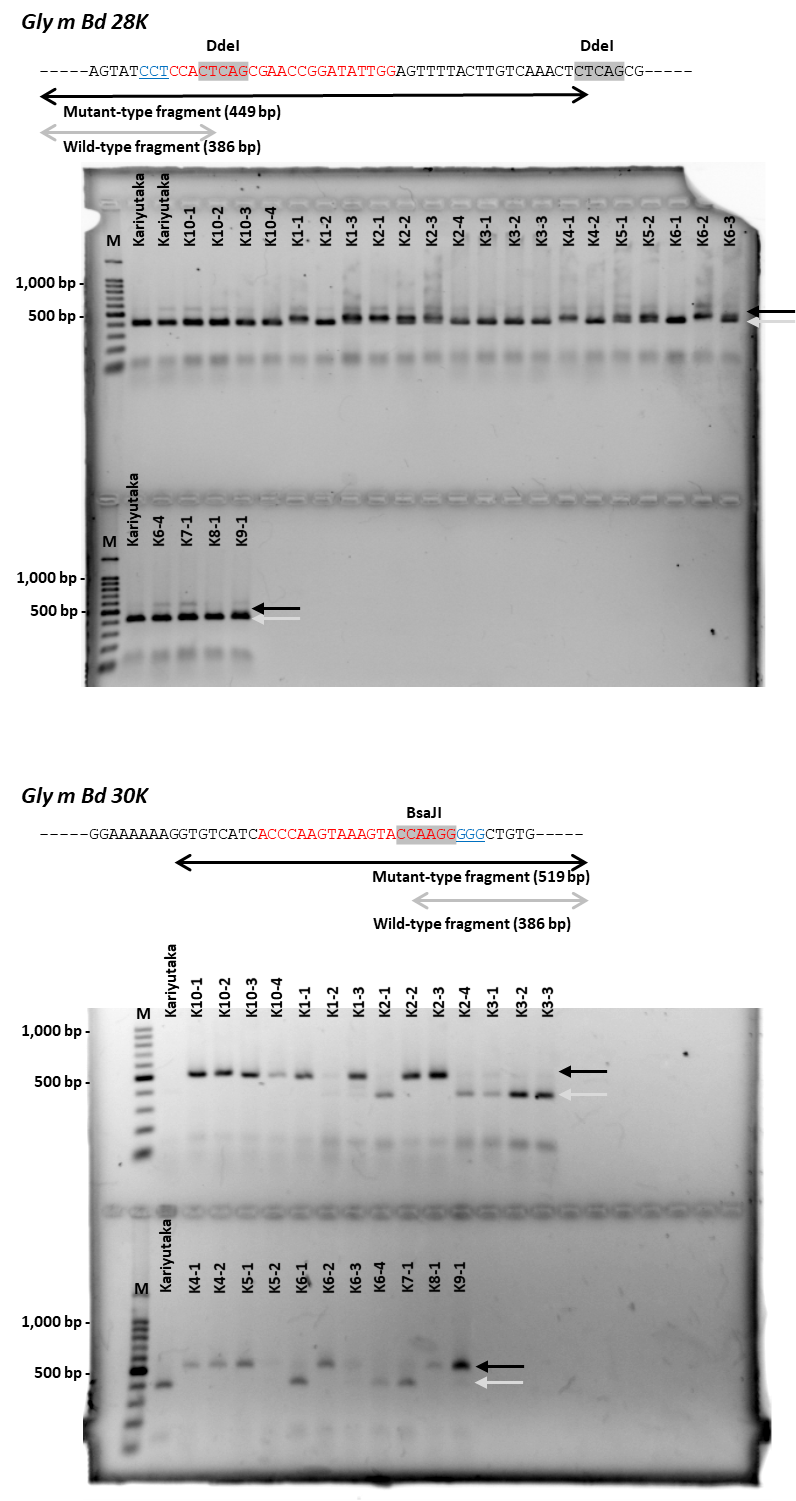


**Figure S1.** Confirmation of mutagenesis of targeted loci in representative Kariuytaka-T_1_ plants by CAPS analysis. The schematic diagrams above panels show DdeI or BsaJI restriction sites (shaded in gray) in fragments amplified with specific primers. Red and underlined blue nucleotide sequences indicate the targeted region of gRNAs designed in this study and PAM, respectively. Gray arrows, expected wild-type fragments; black arrows, expected mutant-type fragments. M, molecular weight marker (100-bp ladder).


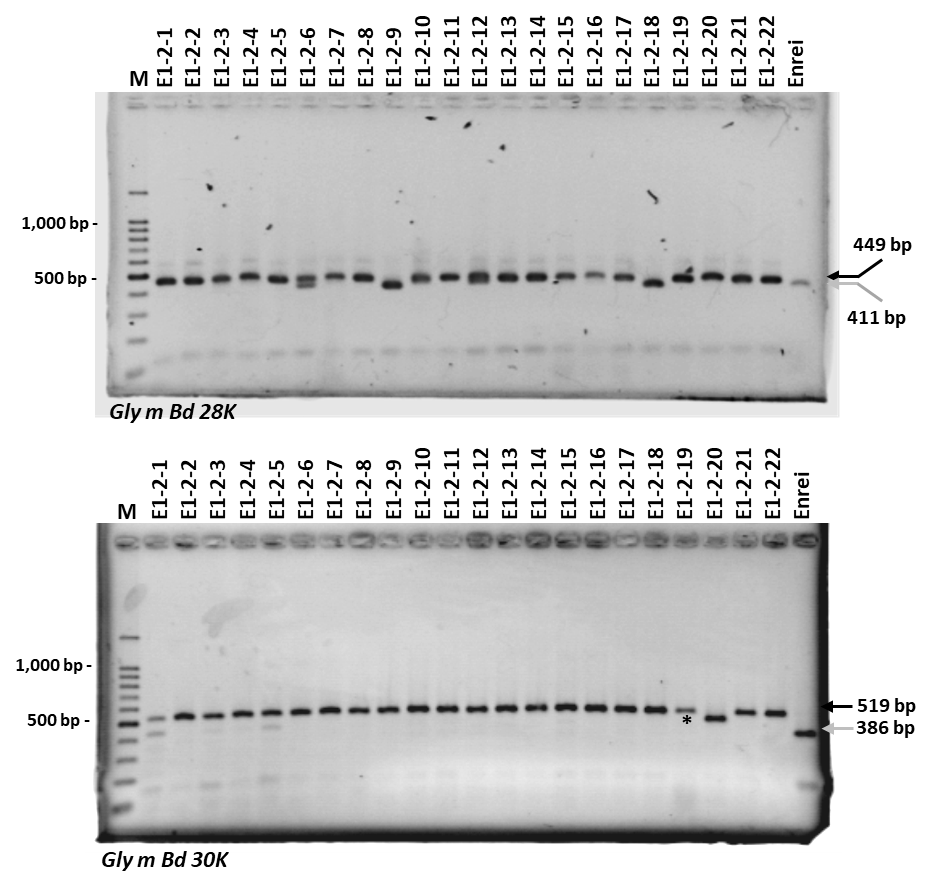


**Figure S2.** Detection of mutations in the *Gly m Bd 28K* and *Gly m Bd 30K* loci in representative Enrei-T_2_ seeds by CAPS analysis. Image denotes agarose gel electrophoresis of fragments. Gray arrows, expected wild-type fragments; black arrows, expected mutant-type fragments. Asterisk, a fragment of unexpected size considered as mutant type. M, molecular weight marker (100-bp ladder).


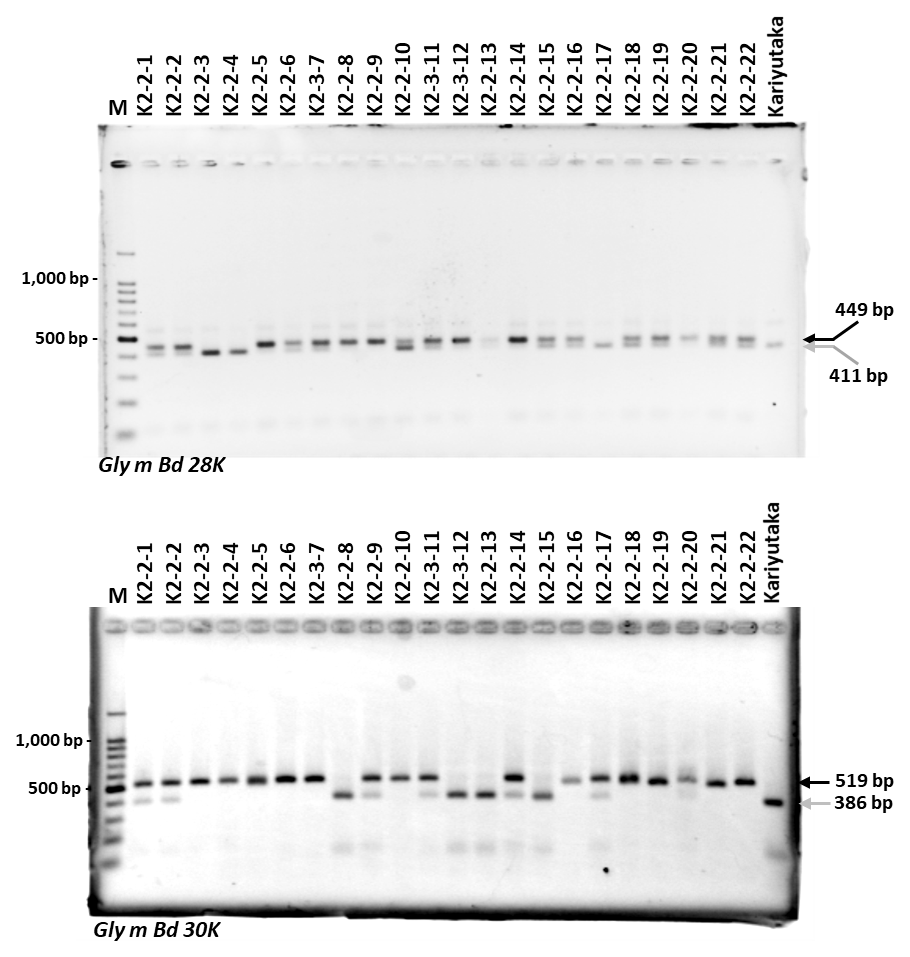


**Figure S3.** Detection of mutations in the *Gly m Bd 28K* and *Gly m Bd 30K* loci in representative Kariyutka-T_2_ seeds by CAPS analysis. Image denotes agarose gel electrophoresis of fragments. Gray arrows, expected wild-type fragments; black arrows, expected mutant-type fragments. M, molecular weight marker (100-bp ladder).


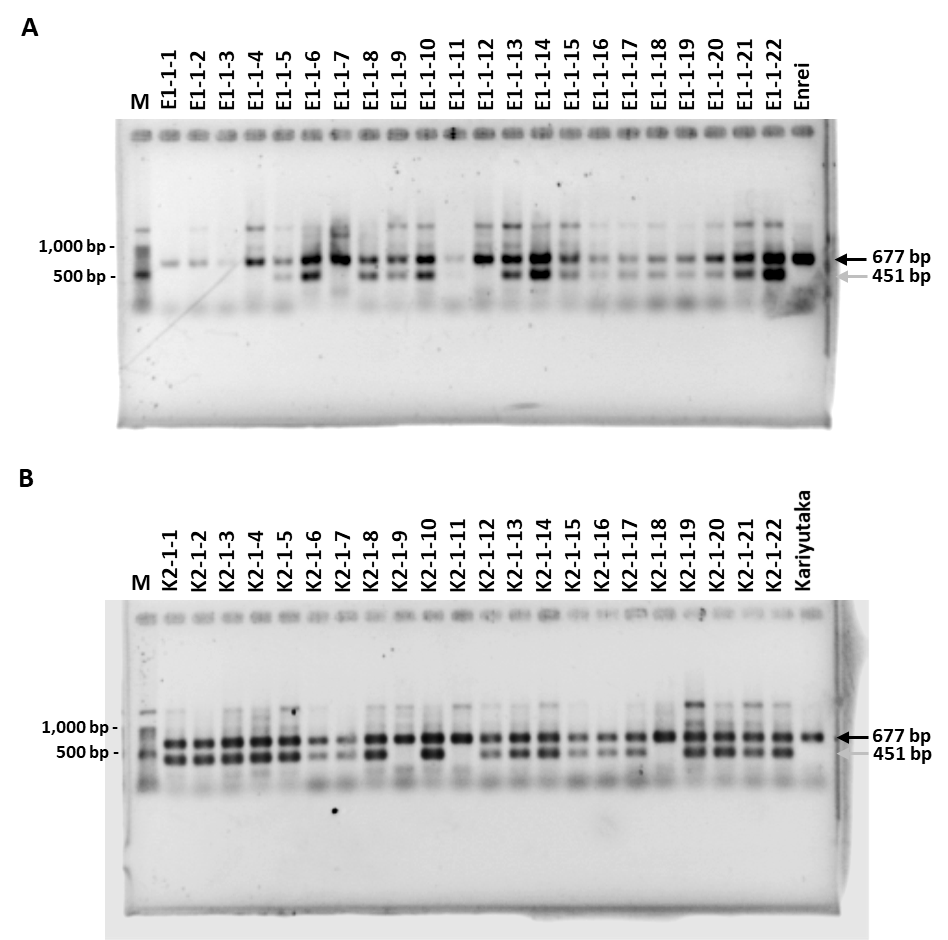


**Figure S4.** Detection of the integration of the *Cas9* gene in representative T_2_ seeds by PCR analysis. (a) Enrei- and (b) Kariyutaka-T_2_ seeds. Black arrows, amplified products of an endogenous gene (Glyma.01G214600) as a positive control; gray arrows, amplified products of *Cas9* gene. M, molecular weight marker (100-bp ladder).


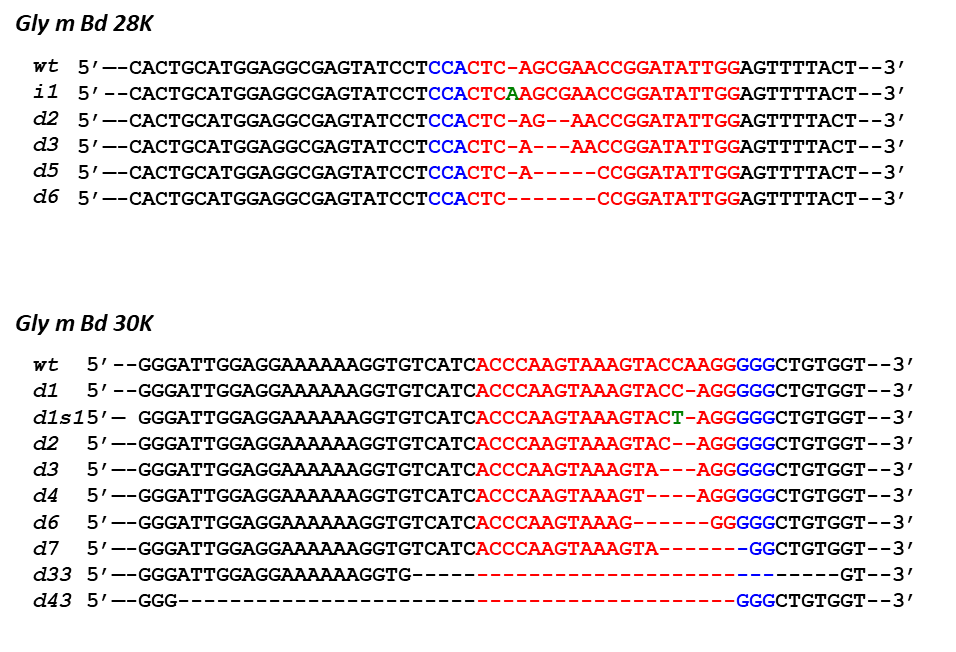


**Figure S5.** Mutational spectra of the targeted loci in double-mutant T_3_ seeds. Red and underlined blue nucleotide sequences indicate the targeted region of gRNAs designed in this study and PAM, respectively. Green nucleotides denote insertion or substitution. Letters and numbers in parentheses indicate the type of mutation in the targeted locus: e.g., *d1*, a single-nucleotide deletion; *i1*, a single-nucleotide insertion; *s1*, a single-nucleotide substitution; *wt*, reference sequences (Enrei or Kariyutaka).


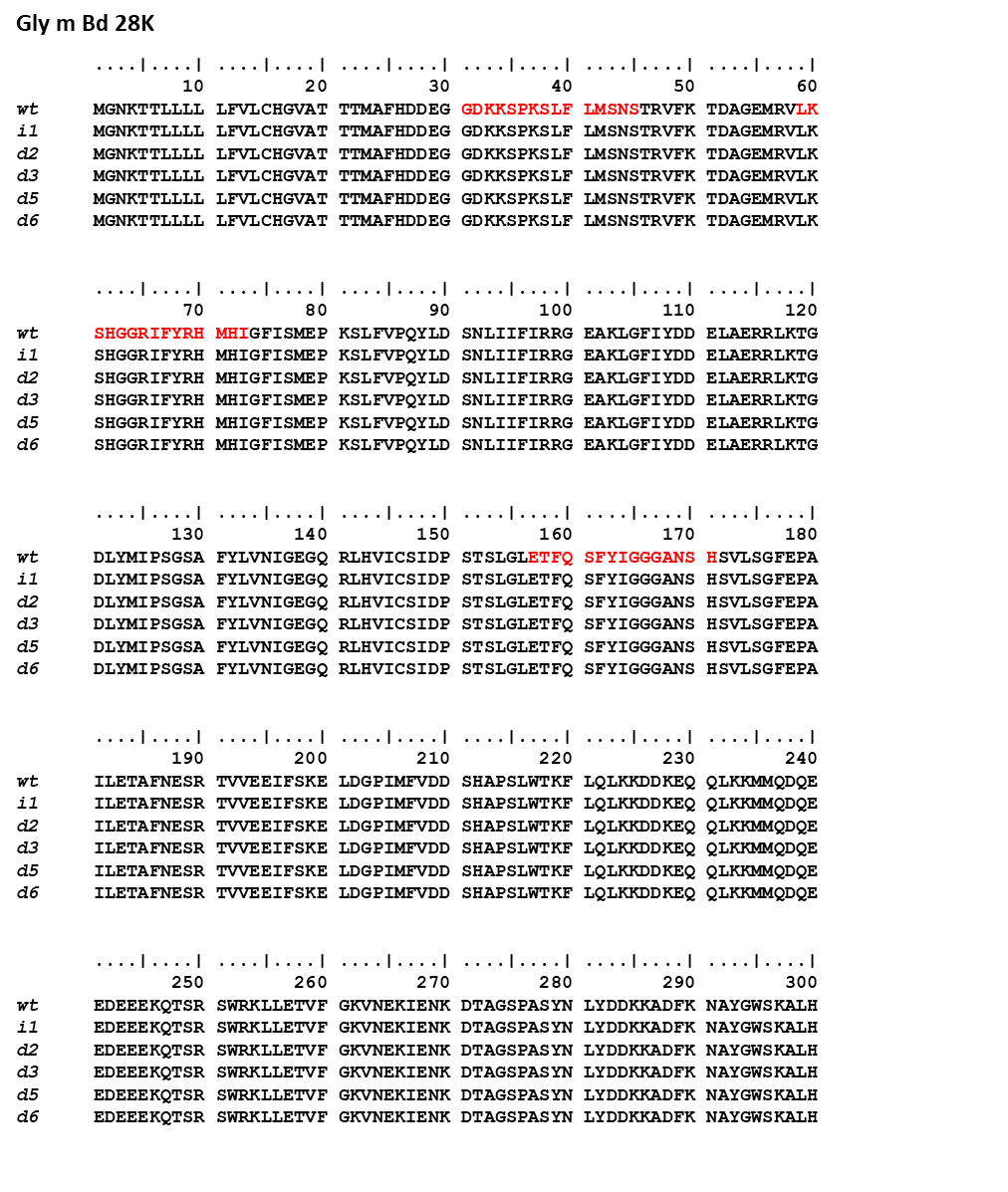


**Figure S6.** Alignment of predicted amino acid sequences of the *Gly m Bd 28K* locus in double mutants.

Letters and numbers in the left side of the alignment denote the type of mutation in the targeted loci; e.g. *d1*, a single-nucleotide deletion; *i1*, a single-nucleotide insertion; *wt*, reference sequence (Enrei or Kariyutaka). Red-colored amino acid residues in the reference sequence indicate epitope regions described in [46].


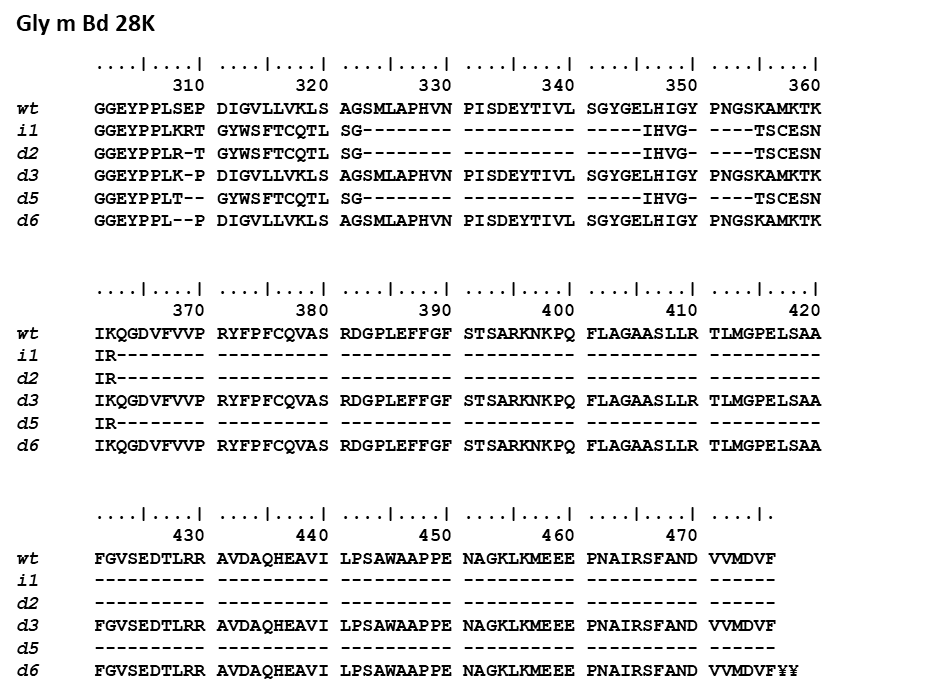


**Figure S6.** Continued


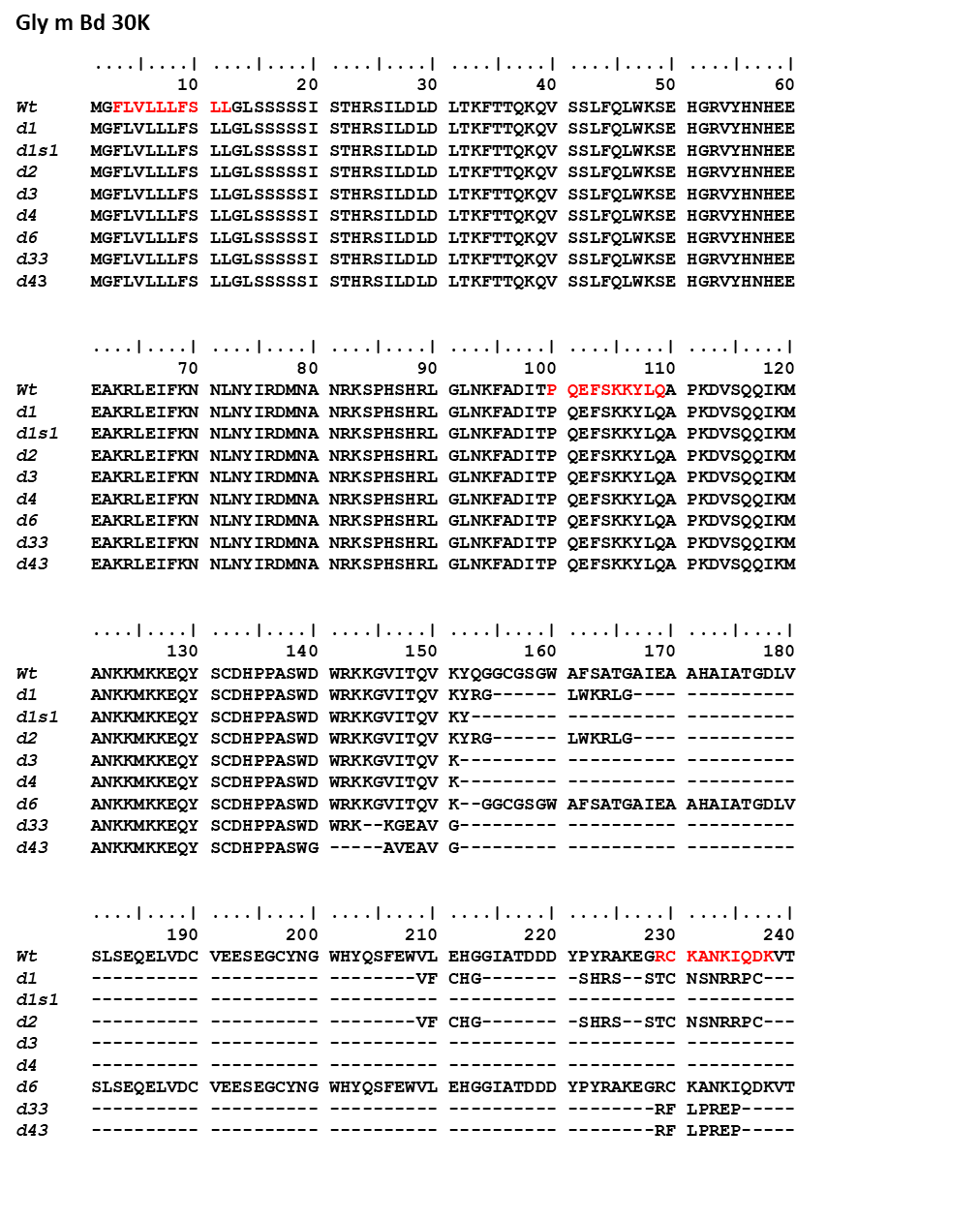


**Figure S7.** Alignment of predicted amino acid sequences of the *Gly m Bd 30K* locus in double mutants. Letters and numbers in the left side of the alignment denote the type of mutation in the targeted loci; e.g. *d1*, a single-nucleotide deletion; *i1*, a single-nucleotide insertion; *s1*, a single-nucleotide substitution; *wt*, reference sequence (Enrei or Kariyutaka). Red-colored amino acid residues in the reference sequence indicate epitope regions described in [44, 45].


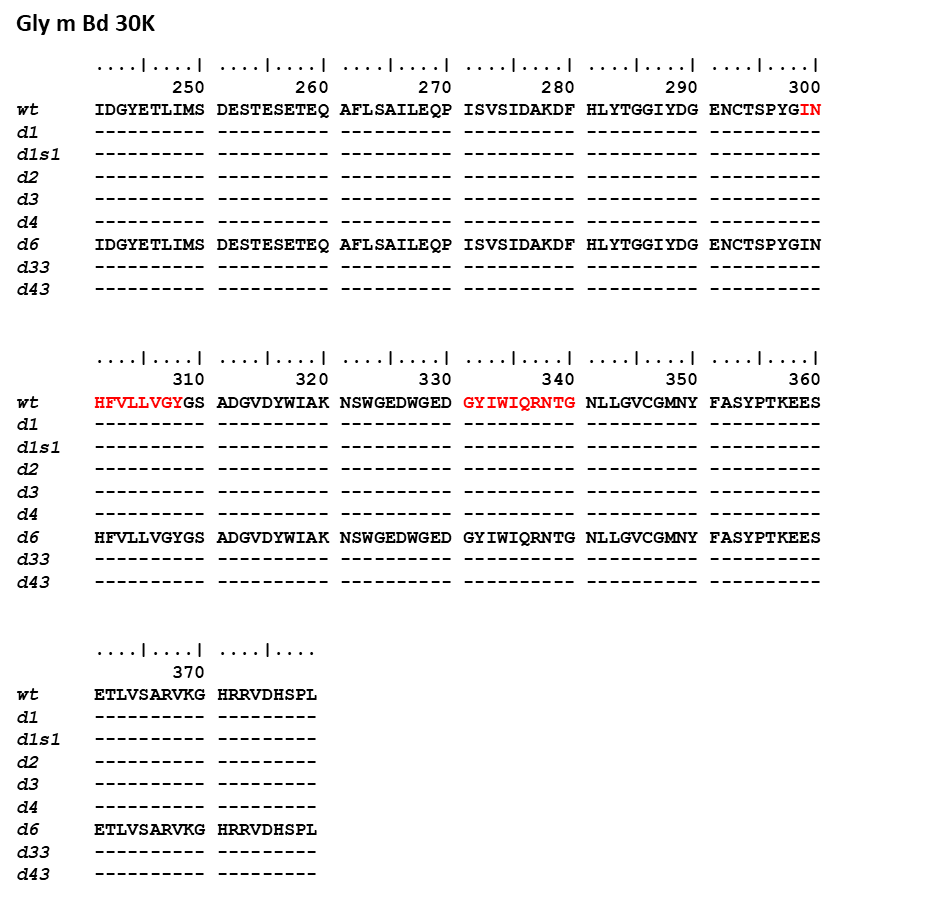


**Figure S7.** Continued


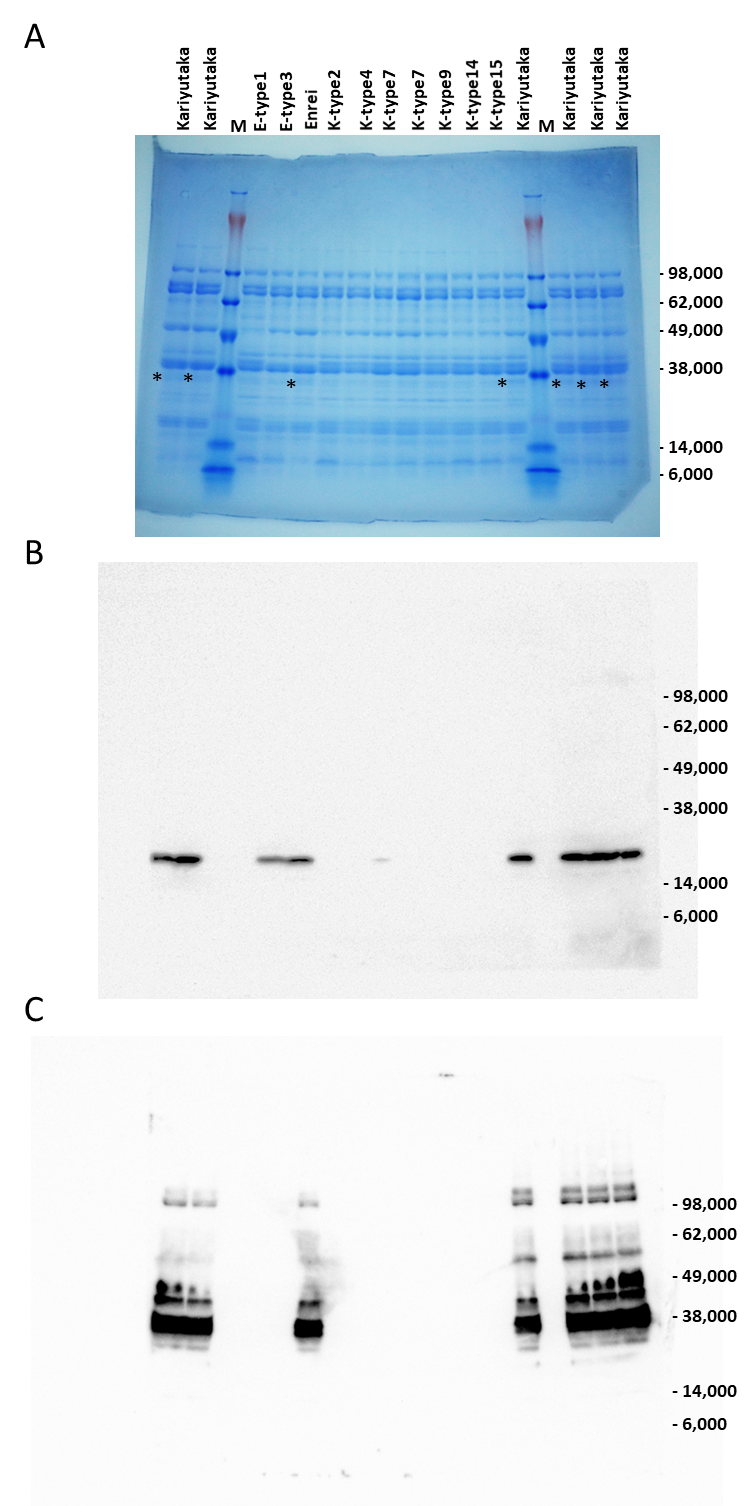


**Figure S8** Full-length gel electrophoresis and immunoblot of the crude protein of representative double-mutant T_3_ and wild-type mature seeds. (a) Proteins separated by SDS-PAGE and stained with Coomasie Brilliant Blue. Designations of mutations are as in Table 3. In parentheses, the left allele refers to *Gly m Bd 28K* and the right one to *Gly m Bd 30K*. Asterisks denote signal of putative Gly m Bd 30K protein. M, molecular weight marker. (b) Immunoblot analysis using polyclonal antibody against Gly m Bd 28K protein. (c) Immunoblot analysis using polyclonal antibody against Gly m Bd 30K protein.


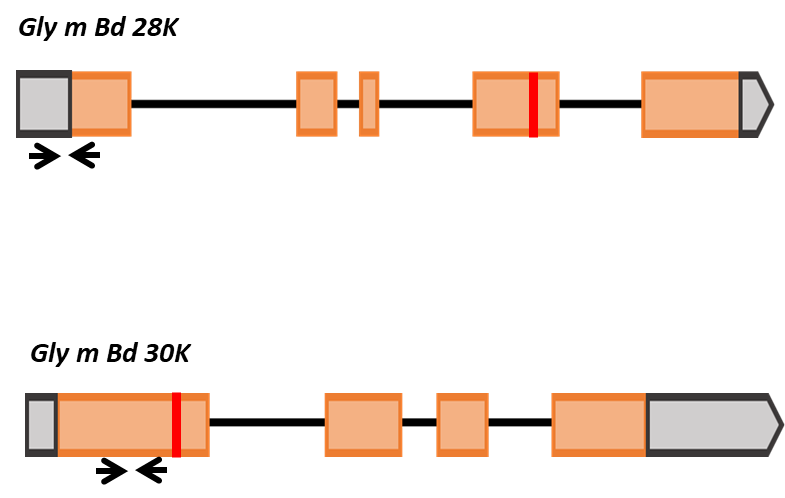


**Figure S9** Primer sites used for semi-quantitative RT-PCR analysis of the *Gly m Bd 30K* and the *Gly m Bd 30K* loci. Boxes and pentagons, exons; bold lines, introns; black arrows, primer regions for semi-quantitative RT-PCR analysis; red lines, sites recognized by the gRNAs designed in this study. Gray- and orange-colored parts denote translated and untranslated regions, respectively.


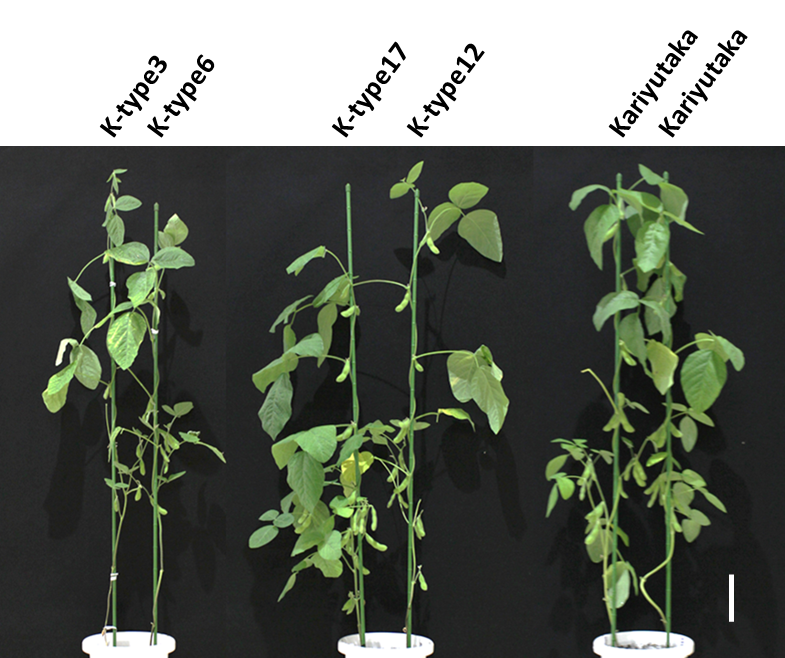


**Figure S10.** Morphological characteristics of representative double-mutant (T_2_) and control Kariyutaka plants. Designations of mutations are equivalent to the haplotypes shown in Table 3. Control plants (Kariyutaka) are grown in the right side pot. Scale bar indicates 10 cm.


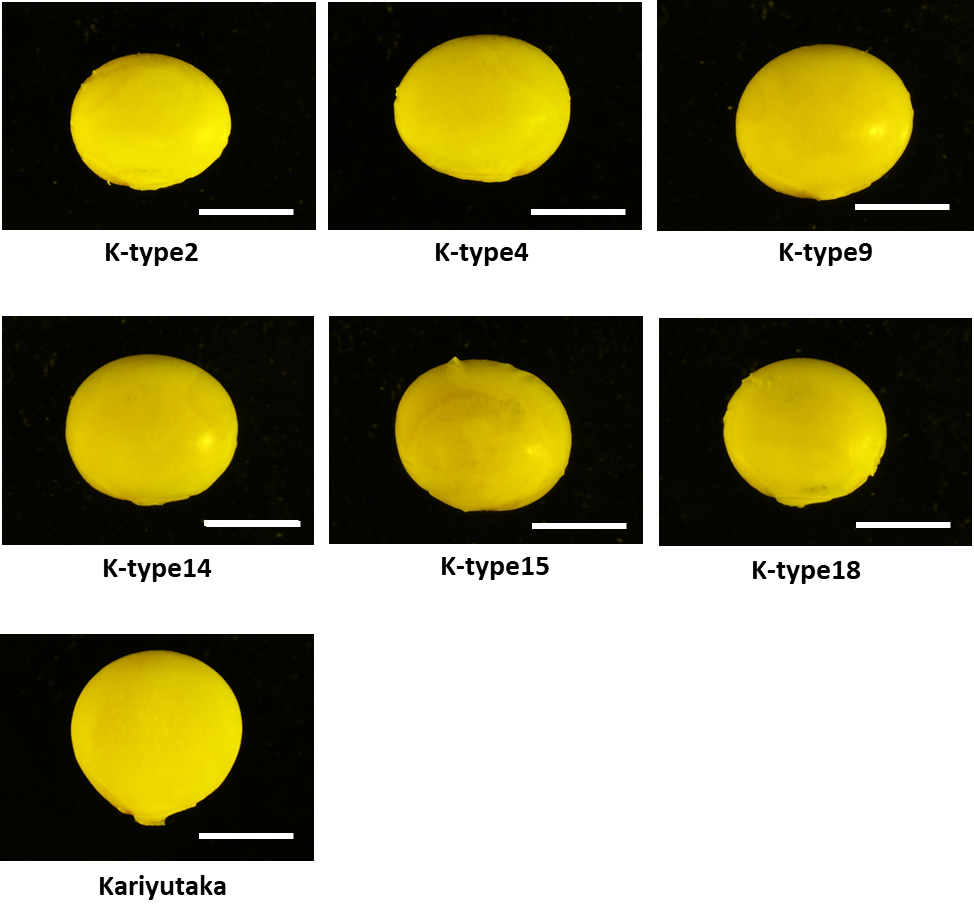


**Figure S11.** Morphological characteristics of representative double-mutant (T_3_) and control Kariyutaka seeds. Designations of mutations are as the haplotypes shown in Table 3. Scale bar indicates 1 cm.
